# Female rats are not more variable than male rats: a meta-analysis of neuroscience studies

**DOI:** 10.1186/s13293-016-0087-5

**Published:** 2016-07-26

**Authors:** Jill B. Becker, Brian J. Prendergast, Jing W. Liang

**Affiliations:** 1 Department of Psychology, Neuroscience Graduate Program, University of Michigan, Ann Arbor, MI USA; 2 Department of Psychiatry, Molecular and Behavioral Neuroscience Institute, University of Michigan, 205 Zina Pitcher Place, Ann Arbor, MI 48109 USA; 3 Department of Psychology, University of Chicago, Chicago, IL USA; 4 Psychology Department, Hunter College, CUNY, New York, NY USA

**Keywords:** Sex differences, Sex bias, Neurobiology, *Rattus norvegicus*

## Abstract

**Background:**

Not including female rats or mice in neuroscience research has been justified due to the variable nature of female data caused by hormonal fluctuations associated with the female reproductive cycle. In this study, we investigated whether female rats are more variable than male rats in scientific reports of neuroscience-related traits.

**Methods:**

PubMed and Web of Science were searched for the period from August 1, 2010, to July 31, 2014, for articles that included both male and female rats and that measured diverse aspects of brain function. Only empirical articles using both male and female gonad-intact adult rats, written in English, and including the number of subjects (or a range) were included. This resulted in 311 articles for analysis. Data were extracted from digital images from article PDFs and from manuscript tables and text. The mean and standard deviation (SD) were determined for each data point and their quotient provided a coefficient of variation (CV) as a measure of trait-specific variability for each sex. Additionally, the results were coded for the type of research being measured (behavior, electrophysiology, histology, neurochemistry, and non-brain measures) and for the strain of rat. Over 6000 data points were extracted for both males and females. Subsets of the data were coded for whether male and female mean values differed significantly and whether animals were grouped or individually housed.

**Results:**

Across all traits, there were no sex differences in trait variability, as indicated by the CV, and there were no sex differences in any of the four neuroscience categories, even in instances in which mean values for males and females were significantly different. Female rats were not more variable at any stage of the estrous cycle than male rats. There were no sex differences in the effect of housing conditions on CV. On one of four measures of non-brain function, females were more variable than males.

**Conclusions:**

We conclude that even when female rats are used in neuroscience experiments without regard to the estrous cycle stage, their data are not more variable than those of male rats. This is true for behavioral, electrophysiological, neurochemical, and histological measures. Thus, when designing neuroscience experiments to include both male and female rats, power analyses based on variance in male measures are sufficient to yield accurate numbers for females as well, even when the estrous cycle is not taken into consideration.

**Electronic supplementary material:**

The online version of this article (doi:10.1186/s13293-016-0087-5) contains supplementary material, which is available to authorized users.

## Background

Twenty years ago, the NIH began requiring all clinical research to include both women and men in research and to report research findings for both sexes. While women are now included in research, fewer women than men are used as subjects, and findings are still not being reported by sex [1]. As a result, any chances for achieving personalized medicine for women in the near future seem remote, as the scientific basis upon which medical decisions are being based are still on data primarily derived from men.

Accounting for sex as a biological variable in all biomedical research is considered fundamental for enhancing rigor and reproducibility in preclinical research [2, 3]. Yet there is considerable concern among preclinical scientists that including female animals will increase costs and variability in data collected [4]. This bias remains entrenched in spite of evidence demonstrating that there are important fundamental biological differences between the sexes, and failure to elucidate these differences is impeding progress in both basic and clinical research [2, 3]. As summarized by Clayton [3, p. 522], “A continual growth in knowledge of the influence of sex at molecular, cellular, and biochemical levels and the various ways that sex exerts influence will inform the design and conduct of additional biomedical research, which is imperative to the NIH mission of turning discovery into health. Understanding scientific findings in the context of sex—be they similarities, differences, and/or complex nuances—is crucial for correctly applying research-derived knowledge toward achieving our ultimate objectives”.

Nevertheless, there is substantial bias in biomedical research to not study female rats or mice and/or to not report the sex of the subjects at all [5–7]. Not including female rats or mice in neuroscience research has been justified due to the variable nature of female data caused by hormonal fluctuations associated with the female’s reproductive cycle, in spite of lack of data in support of this position. A recent meta-analysis reported that female mice are not inherently more variable than male mice across diverse physiological traits [8]. Similar results have been obtained for measures of gene expression in mice and humans [9].

In this study, we investigated whether female and male rats differ in their variability in studies that focused on neuroscience outcomes. We chose to focus on one field for this study in order to examine a dataset that is relatively homogenous, so that failure to find a sex difference in variability would not be due to heterogeneity of the measures being examined. We examined studies that included intact adult male and female rats. The majority of the studies used female rats without regard to the stage of the estrous cycle, but we also examined 26 studies that included male and female rats at specific stages of the estrous cycle. We now report that female rats are not more variable than male rats on studies of neuroscience-related traits. This is true when females are used without regard to the estrous cycle or when studied at specific days of the estrous cycle.

## Methods

### Search strategy

PubMed and Web of Science were searched for the period from August 1, 2010 to July 31, 2014. The PubMed search terms used were as follows: (1) (rat AND gender differences) AND (brain OR neuroscience OR neuron) = 411 articles and (2) (rat AND sex differences) AND (brain OR neuroscience OR neuron) = 525 articles. When these lists were manually combined, this yielded 543 unique articles. On Web of Science, the search terms were TS = (male and female) AND TS = (neuro* AND rat) NOT TS = (adolescent) NOT TS = (mice). These articles were then filtered by neuroscience, behavior, article (not review) and 2010–2014. The Web of Science search generated 743 references; these were manually curated to identify 151 unique additional relevant references using the titles and abstracts (manually eliminated January 1, 2010–July 31, 2010, and any in August 2014). When combined with the PubMed search there were a total of 562 articles. These articles were manually reviewed to determine appropriateness for inclusion. Only empirical articles using both male and female gonad-intact adult rats, written in English, and describing the number of subjects (or a range) were included—resulting in 311 articles for analysis. A list of the articles used is included in the supplemental information for this article (see Additional file 1).

### Data extraction

Data were extracted from digital image files generated from high-resolution screenshots of article PDFs and from manuscript tables and text. Vector graphics software (Adobe Illustrator) was used to quantify the mean and standard deviation (STDEV) or standard error of the mean (SEM) values directly from figure images (in mm), which provided a relative measure of the mean and STDEV/SEM for each data point as described in [8]. Briefly, figures were imported into Adobe Illustrator, and for each data point used, rectangles were positioned on the graphs over the SEM/SD bar from the middle of the data point or bar to the end of the error bar. A rectangle was also positioned from the *X*-axis to the middle of the data point or bar (with corrections if the scale was discontinuous), and the length of each of these rectangles in millimeters (determined by the graphics software) was used as a relative measure of the mean and error reported. Data were only used if the mean and STDEV or SEM could be extracted from the article. Data presented in tables were transcribed directly from the table. For line graphs with more than three time points, values were obtained from the beginning, middle, and end of the time course, so that no one study contributed a disproportionate number of data points to the overall analysis. When a range for the number of subjects was given, the lowest number in the range was used. Data were collected by 10 undergraduate students with an inter-rater reliability coefficient >0.96.

Results were coded for the type of research (behavior, electrophysiology, histology, neurochemistry, and non-brain measures). Behavior was any behavioral measure (*N* = 2245 data points). Electrophysiology included measures of electrical neural activity (LTP, unit activity, cell clamp electrophysiology, etc.; *N* = 364 data points). Neurochemistry was any measure of neurotransmitter or neurotransmitter receptor amount, protein amount, synthesis, second messengers, or neurotransmitter release (*N* = 1809 data points). Most of the molecular studies were included this category. Histology was measures of cellular location, dendritic/axonal branching, brain regions, and brain region activity, including c-fos; measures that quantify physical structure in the brain (*N* = 1233 data points). Non-brain measures (*N* = 601 data points) were any measures of non-central nervous system biology including body weight (*N* = 127 data points), blood/serum hormone measures (*N* = 214 data points), cardio measures (heart rate, blood pressure, etc.; *N* = 54 data points), and blood or organ measurement of exogenous compounds or organ weights (“organ” *N* = 207 data points).

For histology and neurochemistry measures, each pair of data points was also coded for whether male and female values were significantly different from each other. For the histology data, the number of data points each for males and females was as follows: no sex difference = 648 data points; sex difference = 585 data points. For the neurochemistry data, the number of data points each for males and females was as follows: no sex difference = 1177 data points; sex difference = 451 data points; not measured = 181 data points.

In a subset of manuscripts, one or more estrous cycle stages were recorded (*n* = 26 manuscripts). Analysis was without respect to subject category. Not all studies examined all phases of the estrous cycle. We obtained the following number of values: males = 343 data points; diestrus = 330 data points; proestrus = 151 data points; estrus = 241 data points.

For neurochemistry and behavior measures (*n* = 4137 data points, in total), we also evaluated whether the animals were housed individually (*N* = 872 data points; 29 studies), in pairs or two to three/cage (*N* = 1311 data points; 57 studies), three or more per cage (*N* = 1062 data points; 47 studies), or not reported (*N* = 892 data points; 39 studies or 22.6 % of the studies). Housing conditions were the same for males and females in all studies. Thus, the number of data points is the same for both males and females.

The strain of rat was coded when it was indicated in the article (Sprague-Dawley: *N* = 2871 data points; Long-Evans: *N* = 1053 data points; Wistar: *N* = 2221 data points; Norway Brown: *N* = 50 data points).

### Statistical analyses

The coefficient of variation (CV) was calculated as the standard deviation divided by the mean (STDEV/mean) for each data point. Male-female differences were analyzed by paired *t* tests (pairing by data points for male and female collected in an individual study) or analysis of variance (ANOVA; depending on whether individual traits or multiple traits were being compared, respectively). The ANOVAs were followed by pairwise comparisons with Tukey’s multiple comparisons test.

Female to male ratios of CV were calculated to determine if the distribution of variation differed by sex. To calculate, the female to male ratio = [CV female/(CV female + CV male)]. The theoretical mean for the ratios would be 0.5 if males and females did not differ in the coefficient of variability. The CV ratios for each trait were tested for each sex against the theoretical mean by *t* test to examine whether each differed from 0.5.

Inter-rater reliability was determined by Pearson *r* correlation to be 0.960–0.997.

## Results

### Female and male trait variability

**Fig. 1:**
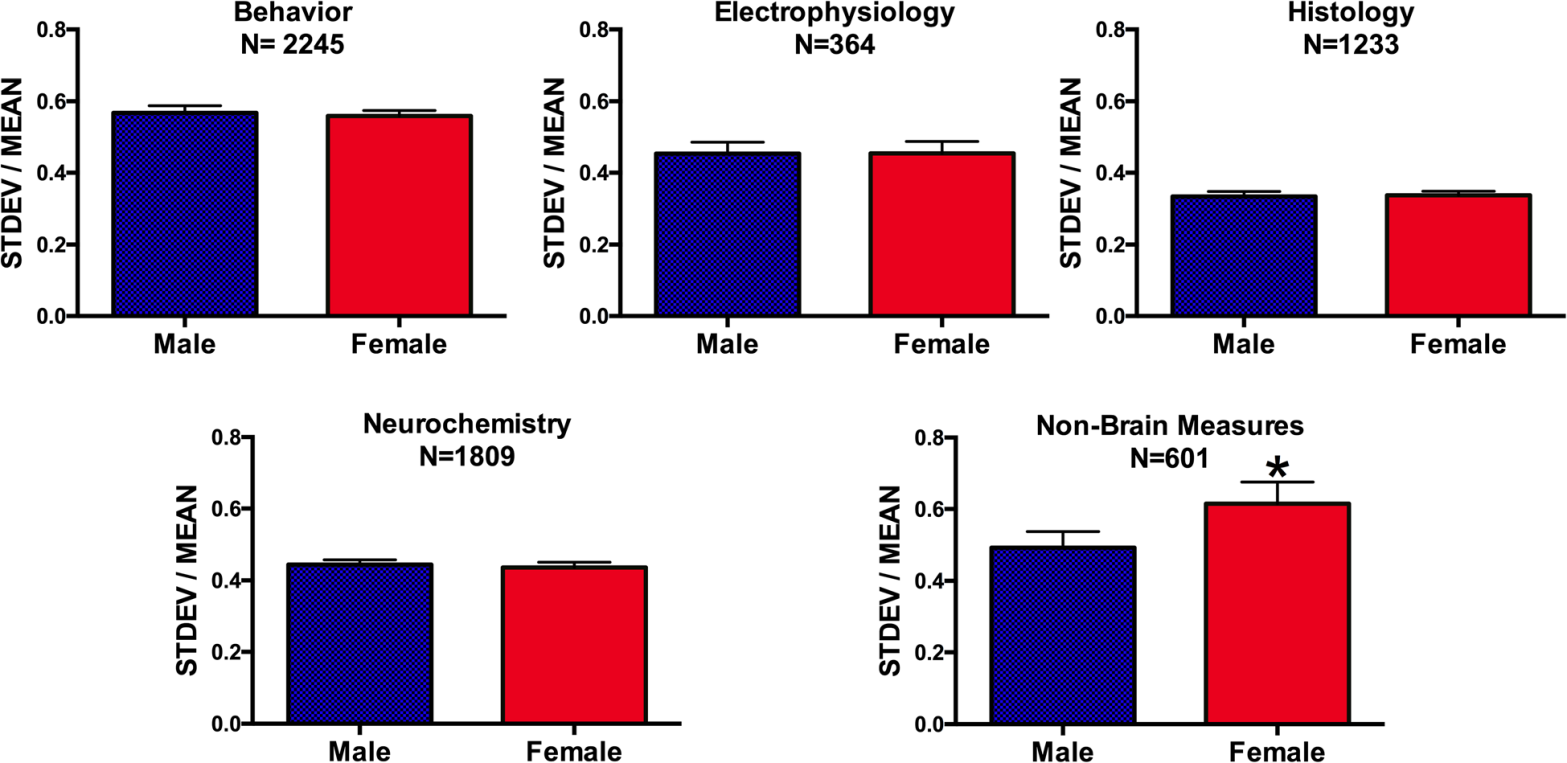
Trait variance as indicated by the standard deviation (STDEV) divided by the mean for behavioral measures, electrophysiological measures, histological measures, and neurochemistry and non-brain measures. *N* = number of data points each for males and females. For “non-brain measures,” there was greater variability for females. *Females > males (*p* = 0.03 on a Mann-Whitney *U* test). SEM indicated by the *lines above the bars*

There were no sex differences in the coefficient of trait variability (CV = STDEV/mean) for any of the neuroscience measures when the CVs for data points obtained from males and females for a given measure from each study were compared with paired *t* tests (Table 1). For behavior, electrophysiology, histology, and neurochemistry data, we found that females were not more variable than males (Fig. 1).

**Table 1:** Individual paired *t* tests comparing males and females on the same measures for each of the trait categories

	*t* value	DF	Number	*p*
Behavior	0.4249	2244	2245	0.6709
Electrophysiology	0.0598	363	364	0.9523
Histology	0.2952	1232	1233	0.7679
Neurochemistry	0.5148	1808	1809	0.6068
Non-brain measures	2.001	600	601	0.0458^a^

^a^Females and males were significantly different on the non-brain measures, but not on any of the other measures

There were, however, differences among the traits in the extent of variability. On a two-way ANOVA (sex X trait), there was no main effect of sex (*F*(1, 12,500) = 1.927; *p* = 0.1651) and no significant sex by trait interaction (*F*(4, 12,500) = 1.574; *p* = 0.1787). There was a main effect of trait (*F*(4, 12,500) = 18.98; *p* < 0.0001) indicating that the CVs for some traits were more variable than other traits. Using Tukey’s multiple comparisons test, the CV for behavior for males was greater than that of histology or neurochemistry (*p* < 0.001), and the CV for histology was lower than that for neurochemistry or non-brain measures (*p* < 0.01). For females, the CV for behavior was also was greater than that of histology or neurochemistry (*p* < 0.001), the CV for histology was lower than the CV for neurochemistry (*p* < 0.01), and the CV for non-brain measures was greater than that for electrophysiology (*p* < 0.05), histology (*p* < 0.001), and neurochemistry (*p* < 0.01). This indicates that even though males and females do not differ from each other, behavioral measures were more variable for both males and females than were neurochemistry and histology measures. On the other hand, histology CV data were less variable for both males and females than neurochemistry or the non-brain measures.

**Fig. 2:**
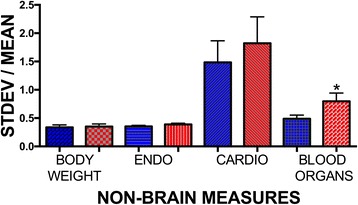
Trait variance as indicated by the standard deviation (STDEV) divided by the mean for non-brain measures further categorized. When sub-categories of non-brain measures were further scrutinized, we found there was greater variability for females only for the blood/organ measures. *Females > males (*p* = 0.036 on a Mann-Whitney *U* test). Males—*blue bars*, females—*red bars*. SEM indicated by the *lines above the bars*

For “non-brain measures,” there was a significant difference when females and males were compared with a paired *t* test (Table 1). The non-brain measures included measures where the mean would be expected to vary with the estrous cycle (body weight, heart rate, blood pressure, organ weights, serum gonadal and adrenal hormones, etc.). To further investigate the source of the variance, we further assigned these measures to sub-categories. These categories were as follows: (1) body weight—body weight/fat weight (*N* = 127); (2) endo—hormone measures (*N* = 214); (3) cardio—blood pressure, heart rate, and other cardiac measures (*N* = 54); and (4) blood/organ—measures of organ weight, organ or blood proteins or exogenous substances, and other organ-specific measures (*N* = 207). As illustrated in Fig. 2, the measures of blood/organ were the primary source of the sex difference in the non-brain measures (*t* = 1.952; DF = 412; *p* = 0.0516; Mann-Whitney *U* test *p* = 0.036).

### Distribution of CV ratios

**Fig. 3:**
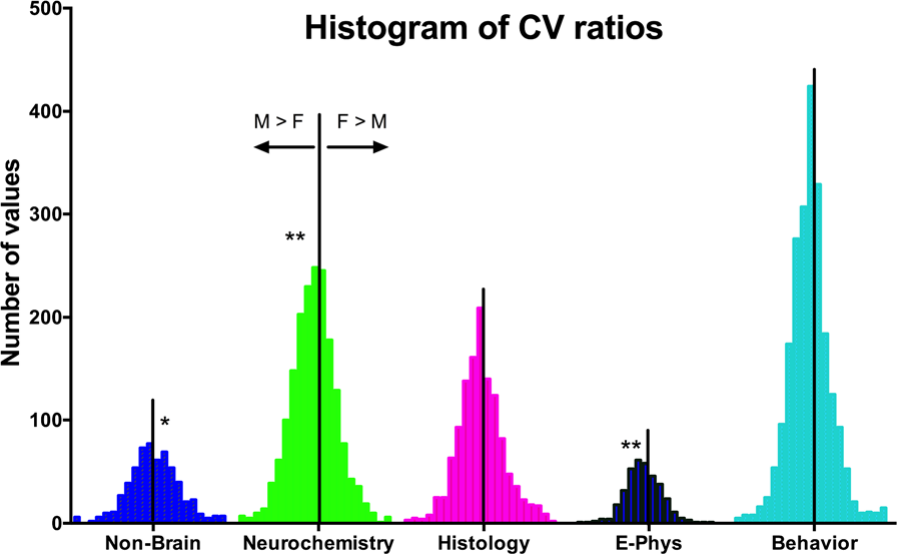
Histogram of distribution of CV ratios (female CV/(female CV + male CV)). To examine whether the variance from the mean was normally distributed for the different traits, we examined the CV ratios. A value of 0.5 (indicated by the *vertical black line*) would indicate that males and females are the same. Values to the *right* of the *vertical black line* for each trait are values where females are more variable than males. Values to the *left* of the *line* indicate males are variable than females. **Males were more variable on the E-Phys trait (*p* = 0.037) and the neurochemistry trait (*p* = 0.0196). *Females were more variable than males on the non-brain measures (*p* < 0.0001)

There was a trend for the distribution of CV ratios (female CV/(female CV + male CV)) to vary by trait on ANOVA (*F* = 2.594, DF = 3, 5650; *p* = 0.0509). As the variance was not normally distributed, the Brown-Forsythe test was considered appropriate to apply and there the analysis indicated that the distribution of CV ratios varied by trait (*F* = 11.91, DF = 3, 5650, *p* < 0.0001; Fig. 3).

We then went on to examine whether there were sex differences in the CV ratios for the different traits. The theoretical mean for the ratios would be 0.5 if males and females did not differ in the CV ratio. When the CV ratios for each trait are tested for each sex against this theoretical mean by *t* test, there was no sex difference in the CV ratio on the behavior (mean = 0.4943 ± 0.0057; *t* = 1.893, DF = 2243) or histology (mean = 0.5050 ± 0.005; *t* = 1.130, DF = 1232) trait categories, and males were more variable than females on the electrophysiology (mean = 0.4863 ± 0.014; *t* = 2.092, DF = 363, *p* = 0.037) and the neurochemistry (mean = 0.4916 ± 0.0084; *t* = 2.336, DF = 1824, *p* = 0.0196) trait categories. Females were more variable than males on the non-brain measures (mean = 0.5308 ± 0.0308, *t* = 4.316, DF = 600, *p* < 0.0001).

### CV values when there is a sex difference in the value

**Fig. 4:**
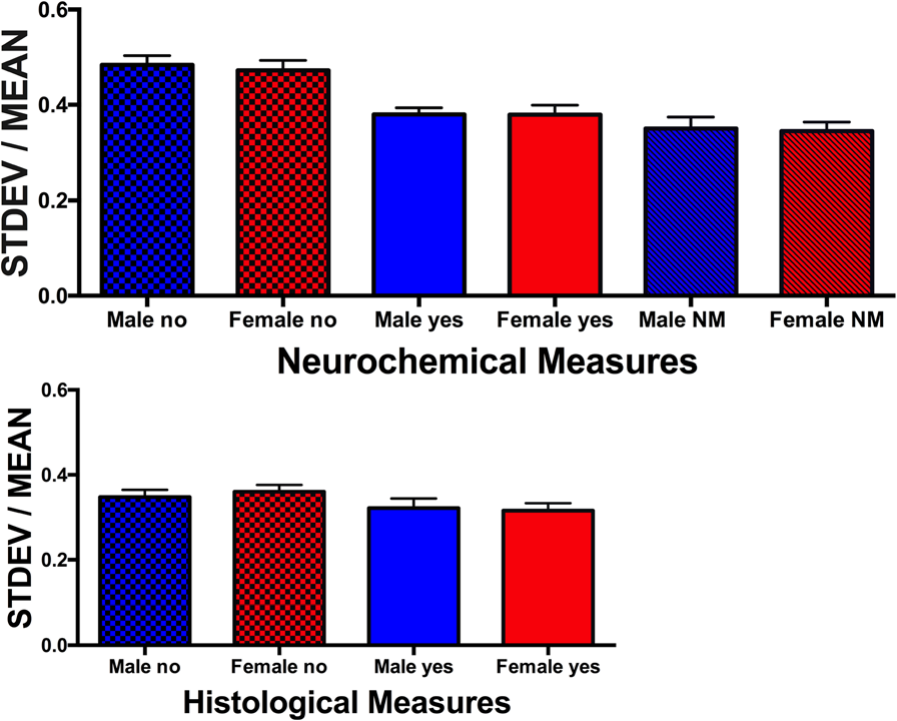
CV values (STDEV/MEAN) for neurochemistry (*top*) and histology (*bottom*) examined based on whether there was a sex difference found for the paired male and female values. CV values did not vary based on whether or not there was a sex difference found. There were only 20 values from the histology articles where a comparison between males and females was not made, so those were excluded. SEM indicated by the *lines above the bars. NM* not measured

We went on to examine whether there were sex differences in CV values if the data points being compared differed significantly between males and females. This analysis examined two trait categories: the histology measures (where the CV ratio distribution did not differ between females and males) and the neurochemistry measures (where CV ratios indicated greater variability in males). As illustrated in Fig. 4, there was no effect of whether a given data point was significantly different between the sexes on the CV values for either histology or neurochemistry. However, in the neurochemistry category, CVs were greater in females when the mean did not differ significantly from those of males as compared to females whose means differed from those of males (Sidak’s multiple comparisons test, *p* < 0.05).

### Impact of estrous cycle on trait variability

**Fig. 5:**
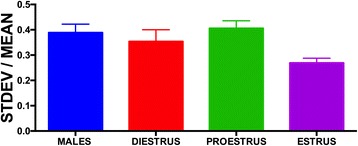
Effect of estrous cycle on sex differences in trait variability. There was no significant effect of estrous cycle or sex differences in trait variability even when phase of the cycle was taken into consideration. SEM indicated by the *lines above the bars*

There was no significant effect of sex/estrous cycle stage on CV with a one-way ANOVA (*F*(3, 1061) = 2.199, *p* = 0.0865; Fig. 5). Females did not differ from males on any day of the estrous cycle nor did any of the female groups differ from each other.

### Impact of housing on trait variability

**Fig. 6:**
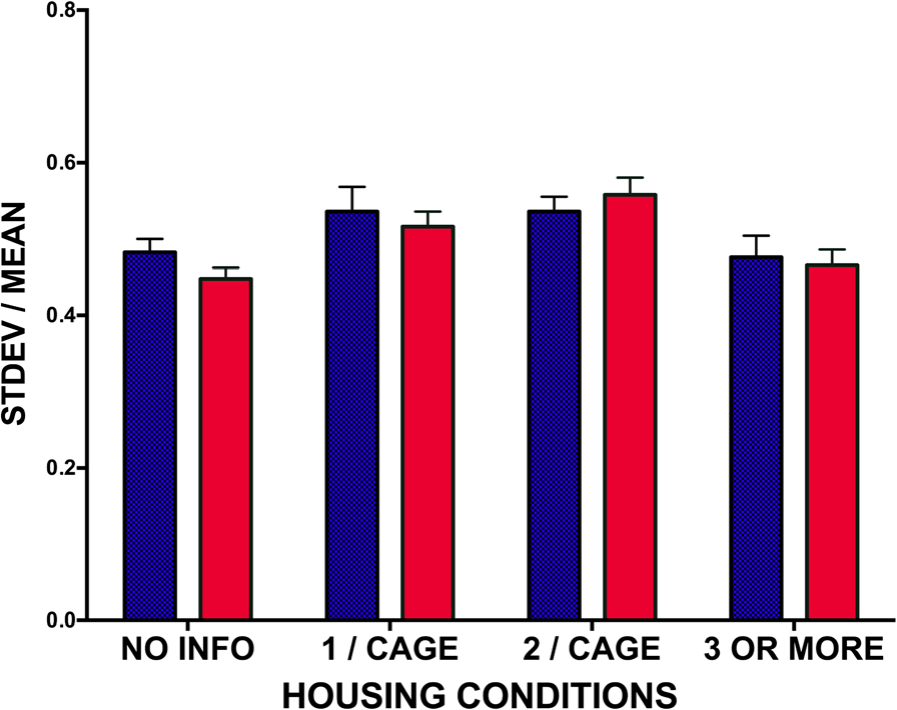
Effect of housing conditions on sex differences in trait variability. There was an overall effect of the number of animals per cage (*p* < 0.0005), but no effect of sex on CV and no interaction. SEM indicated by the *lines above the bars*

For the neurochemistry and behavior values, we also examined whether the housing conditions contributed to the variability in trait data. As can be seen in Fig. 6, there was no sex difference in the effect of housing conditions on trait (*F*(1, 8266) = 0.4282, *p* = 0.5139). Overall, there was a main effect of housing conditions on trait (*F*(3, 8266) = 6.175; *p* = 0.0003), but no sex by trait interaction (*F*(3, 8266) = 0.4282; *p* = 0.5129). These effects do not change if the data from studies where housing conditions were not reported re excluded.

### Impact of rat strain on trait variability

**Fig. 7:**
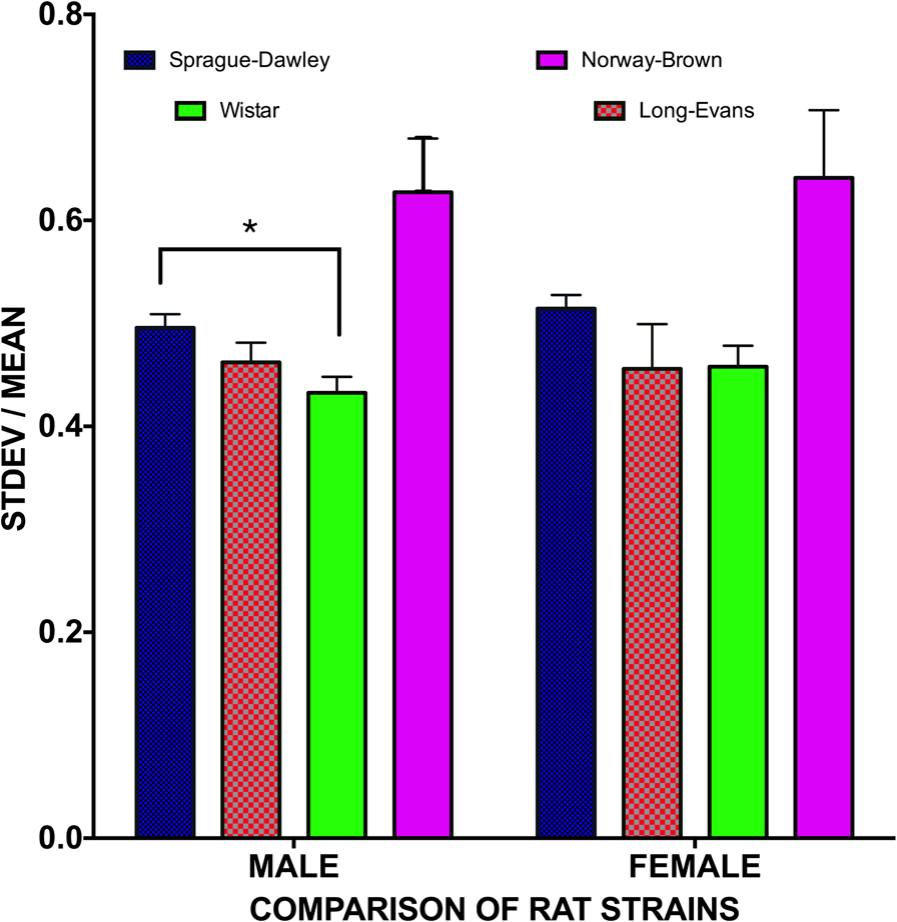
Male Sprague-Dawley rats exhibited greater variance than male Wistar rats *p < 0.05. Sprague-Dawley: *N* = 2871; Long-Evans: *N* = 1053; Wistar: *N* = 2221; Norway Brown: *N* = 50. SEM indicated by the *lines above the bars*

Lastly, we examined whether the strain of rat contributed to variability in data and whether there were effects of sex on the CV; however, there was no effect of strain on sex differences in CV (*F*(1, 12,382) = 0.0889, *p* = 0.765). Overall, male Sprague-Dawley rats were more variable than male Wistar rats (two-way ANOVA; main effect of strain: *F*(3, 12,382) = 3.941; *p* = 0.008; subsequent Tukey’s multiple comparisons test, *p* < 0.05) (see Fig. 7). There were no other effects of strain.

## Discussion

These results indicate that among diverse traits relevant to neuroscience, female rats are no more variable than male rats. When the data are categorized by type of information reported, some types of data have greater intrinsic variability than others: behavioral data are more variable than histology or neurochemistry data, for example; but females and males did not differ in this regard. Thus some types of neuroscience tests may yield more precise, or less variable, data values, but this does not differ by sex. An important and novel aspect of this analysis is that, there were no sex differences evident when males were compared with (1) either randomly cycling females or (2) females at specific, defined stages of the estrous cycle. Moreover, females did not exhibit greater variability at any stage of the estrous cycle, compared with males or with females at other estrous cycle stages.

It is important to note that trait variability was not greater for females or males even when there was a significant sex difference in the mean value reported in the studies analyzed. A significant difference between the sexes on a given measure does not mean that females are more variable than males. What our findings mean is that it is possible to see sex differences in neuroscience studies when equal numbers of male and female rats are used.

There was greater variability among females in the “non-brain” category. Upon further analysis, three of the four defined sub-categories of “non-brain” exhibited no difference whatsoever between males and females. For one indistinct sub-category with a relatively small sample size, there was greater variability in females. Thus, there will be instances where females are more variable than males.

Recently, Itoh and Arnold [9] conducted a meta-analysis of 103 human microarray datasets and 190 mouse microarray datasets to examine gene expression variability in males and females. The results indicated that variability was similar for females and males in humans and in mice and no evidence that female gene expression was more variable than male gene expression in either species. The present report extends the study of sex differences in variability to a species that is widely used in neuroscience and documents the overall absence of sex differences in variability across diverse traits of interest to neuroscientists.

## Conclusions

In conclusion, female rats are not more variable than male rats in neuroscience research. Across a substantially large sampling of research, the data indicate that on average, females exhibit the same (or less) variability on a given trait that male rats do. One implication of these data is that for those investigators initiating research on female rats, power calculations based on data from males would likely be sufficient to determine the number of female subjects needed in order to see a sex difference. There will be particular topics where well-documented effects of the estrous cycle should be considered by investigators in the experimental design in order to get meaningful results. In all datasets, there exists a distribution of CV ratios; thus one single trait may be more variable in males than females (or vice versa). On the other hand, for topics where females have not been studied, these data suggest that inclusion of intact females, without regard to estrous cycle, and intact males is a valid approach to learn about females in neuroscience research.

## Additional file

Additional file 1: Pubmed references used. (DOCX 240 kb)

## Abbreviations

ANOVA: Analysis of variance
CV: Coefficient of variation
SEM: Standard error of the mean
STDEV: Standard deviation
