# Supplementary material for: Female rats are not more variable than male rats: a meta-analysis of neuroscience studies
Source: Biol Sex Differ. 2016 Jul 26;7:34. doi: 10.1186/s13293-016-0087-5 (PMC4962440; doi:10.1186/s13293-016-0087-5)
Supplement: Additional file 1: — Pubmed references used. (DOCX 240 kb) [file 13293_2016_87_MOESM1_ESM.docx]

**PUBMED REFERENCES USED:**

1. Abdel-Sater KA, Abdel-Daiem WM, Sayyed Bakheet M. The gender difference of selective serotonin reuptake inhibitor, fluoxetine in adult rats with stress-induced gastric ulcer. Eur J Pharmacol. 2012 Aug 5;688(1-3):42-8. doi: 10.1016/j.ejphar.2012.04.019. Epub 2012 Apr 21. PubMed PMID: 22546225.
2. Acosta-Rua AJ, Cannon RL, Yezierski RP, Vierck CJ. Sex differences in effects of excitotoxic spinal injury on below-level pain sensitivity. Brain Res. 2011 Oct 24;1419:85-96. doi: 10.1016/j.brainres.2011.08.072. Epub 2011 Sep 5. PubMed PMID:21943508.
3. Adzic M, Lukic I, Mitic M, Djordjevic J, Elakoviƒá I, Djordjevic A, Krstic-Demonacos M, Matiƒá G, Radojcic M. Brain region- and sex-specific modulation of mitochondrial glucocorticoid receptor phosphorylation in fluoxetine treated stressed rats: effects on energy metabolism. Psychoneuroendocrinology. 2013 Dec;38(12):2914-24. doi: 10.1016/j.psyneuen.2013.07.019. Epub 2013 Aug 7.PubMed PMID: 23969420.
4. Admek S, Vyskoƒçil F. Potassium-selective microelectrode revealed difference in threshold potassium concentration for cortical spreading depression in female and male rat brain. Brain Res. 2011 Jan 25;1370:215-9. doi:10.1016/j.brainres.2010.11.018. Epub 2010 Nov 12. PubMed PMID: 21075087.
5. Akhmadeev AV, Kalimullina LB. Paleoamygdala and biogenic amines: effect of gender factor and sex steroid content on their content and metabolism. Bull Exp Biol Med. 2013 Oct;155(6):802-4. PubMed PMID: 24288770.
6. Anderson DW, Pothakos K, Schneider JS. Sex and rearing condition modify theeffects of perinatal lead exposure on learning and memory. Neurotoxicology. 2012 Oct;33(5):985-95. doi: 10.1016/j.neuro.2012.04.016. Epub 2012 Apr 21. PubMedPMID: 22542453; PubMed Central PMCID: PMC3430807.
7. Andrade S, Arbo BD, Batista BA, Neves AM, Branchini G, Brum IS, Barros HM,Gomez R, Ribeiro MF. Effect of progesterone on the expression of GABA(A) receptor subunits in the prefrontal cortex of rats: implications of sex differences and brain hemisphere. Cell Biochem Funct. 2012 Dec;30(8):696-700. doi: 10.1002/cbf.2854. Epub 2012 Jul 16. PubMed PMID: 22806324.
8. Arfaoui A, Lobo MV, Boulbaroud S, Ouichou A, Mesfioui A, Arenas MI. Expression of retinoic acid receptors and retinoid X receptors in normal and vitamin A deficient adult rat brain. Ann Anat. 2013 Mar;195(2):111-21. doi:10.1016/j.aanat.2012.06.006. Epub 2012 Sep 7. PubMed PMID: 23017197.
9. Ashenhurst JR, Seaman M, Jentsch JD. Responding in a test of decision-making under risk is under moderate genetic control in the rat. Alcohol Clin Exp Res. 2012 Jun;36(6):941-9. doi: 10.1111/j.1530-0277.2011.01701.x. Epub 2012 Jan 11.PubMed PMID: 22235982.
10. Atkinson HC, Leggett JD, Wood SA, Castrique ES, Kershaw YM, Lightman SL. Regulation of the hypothalamic-pituitary-adrenal axis circadian rhythm by endocannabinoids is sexually diergic. Endocrinology. 2010 Aug;151(8):3720-7. doi:10.1210/en.2010-0101. Epub 2010 Jun 9. PubMed PMID: 20534730; PubMed Central PMCID: PMC2964781.
11. Ayrance, Altunkaynak BZ, Akta≈ü A, Raƒübetli M√á, Kaplan S. Prenatal exposure of diclofenac sodium affects morphology but not axon number of the median nerve of rats. Folia Neuropathol. 2013;51(1):76-86. PubMed PMID: 23553140.
12. Babb JA, Masini CV, Day HE, Campeau S. Sex differences in activated corticotropin-releasing factor neurons within stress-related neurocircuitry and hypothalamic-pituitary-adrenocortical axis hormones following restraint in rats. Neuroscience. 2013 Mar 27;234:40-52. doi: 10.1016/j.neuroscience.2012.12.051.Epub 2013 Jan 7. PubMed PMID: 23305762; PubMed Central PMCID: PMC3594441.
13. Balkan B, Gozen O, Koylu EO, Keser A, Kuhar MJ, Pogun S. Region- and sex-specific changes in CART mRNA in rat hypothalamic nuclei induced by forced swim stress. Brain Res. 2012 Oct 15;1479:62-71. doi: 10.1016/j. brainres.2012.08.043. Epub 2012 Aug 31. PubMed PMID: 22960117; PubMed Central PMCID: PMC3468742.
14. Bangasser DA, Zhang X, Garachh V, Hanhauser E, Valentino RJ. Sexual dimorphism in locus coeruleus dendritic morphology: a structural basis for sex differences in emotional arousal. Physiol Behav. 2011 Jun 1;103(3-4):342-51. doi:10.1016/j.physbeh.2011.02.037. Epub 2011 Mar 6. PubMed PMID: 21362438; PubMed Central PMCID: PMC3081983.
15. Bangasser DA, Curtis A, Reyes BA, Bethea TT, Parastatidis I, Ischiropoulos H, Van Bockstaele EJ, Valentino RJ. Sex differences in corticotropin-releasing factor receptor signaling and trafficking: potential role in female vulnerability to stress-related psychopathology. Mol Psychiatry. 2010 Sep;15(9):877, 896-904.doi: 10.1038/mp.2010.66. Epub 2010 Jun 15. PubMed PMID: 20548297; PubMed Central PMCID: PMC2935505.
16. Barha CK, Brummelte S, Lieblich SE, Galea LA. Chronic restraint stress in adolescence differentially influences hypothalamic-pituitary-adrenal axis function and adult hippocampal neurogenesis in male and female rats. Hippocampus. 2011 Nov;21(11):1216-27. doi: 10.1002/hipo.20829. Epub 2010 Jul 21. PubMed PMID: 20665592.
17. Baron AW, Rushton SP, Rens N, Morris CM, Blain PG, Judge SJ. Sex differences in effects of low level domoic acid exposure. Neurotoxicology. 2013 Jan;34:1-8.doi: 10.1016/j.neuro.2012.10.010. Epub 2012 Oct 22. PubMed PMID: 23099319.
18. Basso AM, Gallagher KB, Mikusa JP, Rueter LE. Vogel conflict test: sex differences and pharmacological validation of the model. Behav Brain Res. 2011 Mar 17;218(1):174-83. doi: 10.1016/j.bbr.2010.11.041. Epub 2010 Nov 27. PubMedPMID: 21115068.
19. Bayless DW, Darling JS, Stout WJ, Daniel JM. Sex differences in attentional processes in adult rats as measured by performance on the 5-choice serial reaction time task. Behav Brain Res. 2012 Nov 1;235(1):48-54. doi: 10.1016/j.bbr.2012.07.028. Epub 2012 Jul 23. PubMed PMID: 22835820.
20. 10.1097/FBP.0b013e32834eb07d. PubMed PMID: 22129556; PubMed Central PMCID:PMC3303859.
21. Begg DP, Sinclair AJ, Weisinger RS. Reductions in water and sodium intake by aged male and female rats. Nutr Res. 2012 Nov;32(11):865-72. doi:10.1016/j.nutres.2012.09.014. Epub 2012 Oct 25. PubMed PMID: 23176797.
22. Belviranli M, Atalik KE, Okudan N, G√∂kbel H. Age and sex affect spatial and emotional behaviors in rats: the role of repeated elevated plus maze test. Neuroscience. 2012 Dec 27;227:1-9. doi: 10.1016/j.neuroscience.2012.09.036. Epub 2012 Sep 24. PubMed PMID: 23018000.
23. Bender C, de Olmos S, Bueno A, de Olmos J, Lorenzo A. Comparative analyses of the neurodegeneration induced by the non-competitive NMDA-receptor-antagonist drug MK801 in mice and rats. Neurotoxicol Teratol. 2010 Sep-Oct;32(5):542-50.doi: 10.1016/j.ntt.2010.05.002. Epub 2010 May 12. PubMed PMID: 20470881.
24. Bessinis DP, Dalla C, Daifoti ZP, Tiligada E. Histamine involvement in visual development and adaptation. Invest Ophthalmol Vis Sci. 2012 Nov1;53(12):7498-503. doi: 10.1167/iovs.12-10809. PubMed PMID: 23060140.
25. Betharia S, Maher TJ. Neurobehavioral effects of lead and manganese individually and in combination in developmentally exposed rats. Neurotoxicology. 2012 Oct;33(5):1117-27. doi: 10.1016/j.neuro.2012.06.002. Epub 2012 Jun 23.PubMed PMID: 22732189.
26. Blaze J, Scheuing L, Roth TL. Differential methylation of genes in the medial prefrontal cortex of developing and adult rats following exposure to maltreatment or nurturing care during infancy. Dev Neurosci. 2013;35(4):306-16. doi: 10.1159/000350716. Epub 2013 Jun 8. PubMed PMID: 23751776; PubMed Central PMCID: PMC3847900.
27. Bobzean SA, Dennis TS, Addison BD, Perrotti LI. Influence of sex on reinstatement of cocaine-conditioned place preference. Brain Res Bull. 2010 Nov 20;83(6):331-6. doi: 10.1016/j.brainresbull.2010.09.003. Epub 2010 Sep 22. PubMed PMID: 20851744.
28. Boix J, Cauli O, Leslie H, Felipo V. Differential long-term effects of developmental exposure to polychlorinated biphenyls 52, 138 or 180 on motor activity and neurotransmission. Gender dependence and mechanisms involved. Neurochem Int. 2011 Jan;58(1):69-77. doi: 10.1016/j.neuint.2010.10.014. Epub 2010 Nov 5. PubMed PMID: 21056608.
29. Bonn M, Schmitt A, Lesch KP, Van Bockstaele EJ, Asan E. Serotonergic innervation and serotonin receptor expression of NPY-producing neurons in the rat lateral and basolateral amygdaloid nuclei. Brain Struct Funct. 2013 Mar;218(2):421-35. doi: 10.1007/s00429-012-0406-5. Epub 2012 Apr 17. PubMed PMID:22527118; PubMed Central PMCID: PMC3580143.
30. Brunton PJ, Donadio MV, Russell JA. Sex differences in prenatally programmed anxiety behaviour in rats: differential corticotropin-releasing hormone receptor mRNA expression in the amygdaloid complex. Stress. 2011 Nov;14(6):634-43. doi:10.3109/10253890.2011.604750. Epub 2011 Aug 19. PubMed PMID: 21854167.
31. MW, See RE. Corticotrophin releasing factor (CRF) induced reinstatement of cocaine seeking in male and female rats. Physiol Behav. 2012 Jan 18;105(2):209-14. doi: 10.1016/j.physbeh.2011.08.020. Epub 2011 Aug 24. PubMed PMID: 21889522; PubMed Central PMCID: PMC3225499.
32. Bulfin LJ, Clarke MA, Buller KM, Spencer SJ. Anxiety and hypothalamic-pituitary-adrenal axis responses to psychological stress are attenuated in male rats made lean by large litter rearing. Psychoneuroendocrinology. 2011 Aug;36(7):1080-91. doi:10.1016/j.psyneuen.2011.01.006. Epub 2011 Feb 23. PubMed PMID: 21349647.
33. Burke NN, Llorente R, Marco EM, Tong K, Finn DP, Viveros MP, Roche M. Maternal deprivation is associated with sex-dependent alterations in nociceptive behavior and neuroinflammatory mediators in the rat following peripheral nerveinjury. J Pain. 2013 Oct;14(10):1173-84. doi: 10.1016/j.jpain.2013.05.003. Epub2013 Jul 11. PubMed PMID: 23850096.
34. Burstein SR, Williams TJ, Lane DA, Knudsen MG, Pickel VM, McEwen BS, Waters EM, Milner TA. The influences of reproductive status and acute stress on the levels of phosphorylated delta opioid receptor immunoreactivity in rat hippocampus. Brain Res. 2013 Jun 26;1518:71-81. doi: 10.1016/j.brainres.2013.03.051. Epub 2013 Apr 11. PubMed PMID: 23583481; PubMed Central PMCID: PMC3764923.
35. Burton CL, Fletcher PJ. Age and sex differences in impulsive action in rats: the role of dopamine and glutamate. Behav Brain Res. 2012 Apr 21;230(1):21-33.doi: 10.1016/j.bbr.2012.01.046. Epub 2012 Feb 1. PubMed PMID: 22326372.
36. Bychkov E, Ahmed MR, Gurevich EV. Sex differences in the activity of signalling pathways and expression of G-protein-coupled receptor kinases in the neonatal ventral hippocampal lesion model of schizophrenia. Int J Neuropsychopharmacol. 2011 Feb;14(1):1-15. doi: 10.1017/S1461145710000118. Epub2010 Feb 17. PubMed PMID: 20158934; PubMed Central PMCID: PMC2992801.
37. Byrnes JJ, Babb JA, Scanlan VF, Byrnes EM. Adolescent opioid exposure in female rats: transgenerational effects on morphine analgesia and anxiety-like behavior in adult offspring. Behav Brain Res. 2011 Mar 17;218(1):200-5. doi:10.1016/j.bbr.2010.11.059. Epub 2010 Dec 5. PubMed PMID: 21138744; PubMed Central PMCID: PMC3022078.
38. Carbone DL, Zuloaga DG, Hiroi R, Foradori CD, Legare ME, Handa RJ. Prenatal dexamethasone exposure potentiates diet-induced hepatosteatosis and decreases plasma IGF-I in a sex-specific fashion. Endocrinology. 2012 Jan;153(1):295-306. doi: 10.1210/en.2011-1601. Epub 2011 Nov 8. PubMed PMID: 22067322; PubMed Central PMCID: PMC3249671.
39. Carbone DL, Zuloaga DG, Lacagnina AF, McGivern RF, Handa RJ. Exposure to dexamethasone during late gestation causes female-specific decreases in core body temperature and prepro-thyrotropin-releasing hormone expression in the paraventricular nucleus of the hypothalamus in rats. Physiol Behav. 2012 Dec 25;108:6-12. doi: 10.1016/j.physbeh.2012.07.010. Epub 2012 Aug 2. PubMed PMID: 22884559; PubMed Central PMCID: PMC3513498.
40. Carrier N, Kabbaj M. Sex differences in the antidepressant-like effects of ketamine. Neuropharmacology. 2013 Jul;70:27-34. doi: 10.1016/j.neuropharm.2012.12.009. Epub 2013 Jan 19. PubMed PMID: 23337256.
41. Carrier N, Kabbaj M. Sex differences in social interaction behaviors in rats are mediated by extracellular signal-regulated kinase 2 expression in the medial prefrontal cortex. Neuroscience. 2012 Jun 14;212:86-92. doi:10.1016/j.neuroscience.2012.03.041. Epub 2012 Apr 18. PubMed PMID: 22521590;PubMed Central PMCID: PMC3367089.
42. Caruso D, Pesaresi M, Maschi O, Giatti S, Garcia-Segura LM, Melcangi RC.Effect of short-and long-term gonadectomy on neuroactive steroid levels in thecentral and peripheral nervous system of male and female rats. J Neuroendocrinol.2010 Nov;22(11):1137-47. doi: 10.1111/j.1365-2826.2010.02064.x. PubMed PMID:20819120.
43. Carvalho-Netto EF, Myers B, Jones K, Solomon MB, Herman JP. Sex differences in synaptic plasticity in stress-responsive brain regions following chronic variable stress. Physiol Behav. 2011 Aug 3;104(2):242-7. doi: 10.1016/j.physbeh.2011.01.024. Epub 2011 Feb 17. PubMed PMID: 21315096.
44. Cauli O, Piedrafita B, Llansola M, Felipo V. Gender differential effects of developmental exposure to methyl-mercury, polychlorinated biphenyls 126 or 153, or its combinations on motor activity and coordination. Toxicology. 2013 Sep 6;311(1-2):61-8. doi: 10.1016/j.tox.2012.11.016. Epub 2012 Dec 5. PubMed PMID:23220684.
45. Chauvel V, Vamos E, Pardutz A, Vecsei L, Schoenen J, Multon S. Effect of systemic kynurenine on cortical spreading depression and its modulation by sex hormones in rat. Exp Neurol. 2012 Aug;236(2):207-14. doi: 10.1016/j.expneurol.2012.05.002. Epub 2012 May 14. PubMed PMID: 22587906.
46. Chisholm NC, Kim T, Juraska JM. Males, but not females, lose tyrosine hydroxylase fibers in the medial prefrontal cortex and are impaired on a delayed alternation task during aging. Behav Brain Res. 2013 Apr 15;243:239-46. doi:10.1016/j.bbr.2013.01.009. Epub 2013 Jan 15. PubMed PMID: 23327742; PubMedCentral PMCID: PMC3594341.
47. Chow C, Epp JR, Lieblich SE, Barha CK, Galea LA. Sex differences in neurogenesis and activation of new neurons in response to spatial learning and memory. Psychoneuroendocrinology. 2013 Aug;38(8):1236-50. doi:10.1016/j.psyneuen.2012.11.007. Epub 2012 Dec 6. PubMed PMID: 23219473.
48. Cohen OS, Varlinskaya EI, Wilson CA, Glatt SJ, Mooney SM. Acute prenatalexposure to a moderate dose of valproic acid increases social behavior and alters gene expression in rats. Int J Dev Neurosci. 2013 Dec;31(8):740-50. doi:10.1016/j.ijdevneu.2013.09.002. Epub 2013 Sep 19. PubMed PMID: 24055786; PubMedCentral PMCID: PMC3870582.
49. Cory-Slechta DA, Weston D, Liu S, Allen JL. Brain hemispheric differences in the neurochemical effects of lead, prenatal stress, and the combination and their amelioration by behavioral experience. Toxicol Sci. 2013 Apr;132(2):419-30. doi: 10.1093/toxsci/kft015. Epub 2013 Jan 28. PubMed PMID: 23358193; PubMed CentralPMCID: PMC3693514.
50. Cost KT, Williams-Yee ZN, Fustok JN, Dohanich GP. Sex differences in object-in-place memory of adult rats. Behav Neurosci. 2012 Jun;126(3):457-64.doi: 10.1037/a0028363. PubMed PMID: 22642887.
51. Cox BM, Young AB, See RE, Reichel CM. Sex differences in methamphetamine seeking in rats: impact of oxytocin. Psychoneuroendocrinology. 2013 Oct;38(10):2343-53. doi: 10.1016/j.psyneuen.2013.05.005. Epub 2013 Jun 12. PubMed PMID: 23764194; PubMed Central PMCID: PMC3775911.
52. Damborsky JC, Griffith WH, Winzer-Serhan UH. Chronic neonatal nicotineexposure increases excitation in the young adult rat hippocampus in a sex-dependent manner. Brain Res. 2012 Jan 9;1430:8-17. doi:10.1016/j.brainres.2011.10.039. Epub 2011 Nov 4. PubMed PMID: 22119395; PubMed Central PMCID: PMC3260652.
53. Desgent S, Duss S, Sanon NT, Lema P, Levesque M, Hubert D, R√©billard RM, Bibeau K, Brochu M, Carmant L. Early-life stress is associated with gender-based vulnerability to epileptogenesis in rat pups. PLoS One. 2012;7(8):e42622. doi:10.1371/journal.pone.0042622. Epub 2012 Aug 3. PubMed PMID: 22880055; PubMedCentral PMCID: PMC3411822.
54. Dickerson SM, Cunningham SL, Patisaul HB, Woller MJ, Gore AC. Endocrine disruption of brain sexual differentiation by developmental PCB exposure. Endocrinology. 2011 Feb;152(2):581-94. doi: 10.1210/en.2010-1103. Epub 2010 Dec 29. PubMed PMID: 21190954; PubMed Central PMCID: PMC3037168.
55. DuBois B, Pearson J, Hastings B, Mahmood T, Chan T, Alnakhli A, Cherala G. Maternal low-protein diet alters the expression of real-time quantitative polymerase chain reaction reference genes in an age-, sex-, and organ-dependent manner in rat offspring. Nutr Res. 2013 Mar;33(3):235-41. doi:10.1016/j.nutres.2013.01.003. Epub 2013 Feb 4. PubMed PMID: 23507230.
56. Dumais KM, Bredewold R, Mayer TE, Veenema AH. Sex differences in oxytocin receptor binding in forebrain regions: correlations with social interest in brain region- and sex- specific ways. Horm Behav. 2013 Sep;64(4):693-701. doi:10.1016/j.yhbeh.2013.08.012. Epub 2013 Sep 18. PubMed PMID: 24055336.
57. Ericson M, Norrsj√∂ G, Svensson AI. Behavioral sensitization to nicotine in female and male rats. J Neural Transm. 2010 Sep;117(9):1033-9. doi:10.1007/s00702-010-0449-9. Epub 2010 Aug 7. PubMed PMID: 20694487.
58. Feinstein I, Kritzer MF. Acute N-methyl-D-aspartate receptor hypofunction induced by MK801 evokes sex-specific changes in behaviors observed in open-field testing in adult male and proestrus female rats. Neuroscience. 2013 Jan 3;228:200-14. doi: 10.1016/j.neuroscience.2012.10.026. Epub 2012 Oct 22. PubMed PMID: 23085219; PubMed Central PMCID: PMC3525798.
59. Feng X, Zhang T, Ralston E, Ludlow CL. Differences in neuromuscular junctions of laryngeal and limb muscles in rats. Laryngoscope. 2012 May;122(5):1093-8. doi: 10.1002/lary.23218. Epub 2012 Feb 28. PubMed PMID:22374515; PubMed Central PMCID: PMC3462430.
60. Ferhatovic L, Banozic A, Kostic S, Sapunar D, Puljak L. Sex differences in pain-related behavior and expression of calcium/calmodulin-dependent protein kinase II in dorsal root ganglia of rats with diabetes type 1 and type 2. Acta Histochem. 2013 Jun;115(5):496-504. doi: 10.1016/j.acthis.2012.11.006. Epub 2012 Dec 23. PubMed PMID: 23267764.
61. Francis-Oliveira J, Ponte B, Barbosa AP, Ver√≠ssimo LF, Gomes MV, Pelosi GG, Britto LR, Moreira EG. Fluoxetine exposure during pregnancy and lactation: Effects on acute stress response and behavior in the novelty-suppressed feeding are age and gender-dependent in rats. Behav Brain Res. 2013 Sep 1;252:195-203. doi: 10.1016/j.bbr.2013.05.064. Epub 2013 Jun 10. PubMed PMID: 23764459.
62. Friedman J, Frye C. Anti-anxiety, cognitive, and steroid biosynthetic effects of an isoflavone-based dietary supplement are gonad and sex-dependent in rats. Brain Res. 2011 Mar 16;1379:164-75. doi: 10.1016/j.brainres.2010.12.025.Epub 2010 Dec 15. PubMed PMID: 21167133; PubMed Central PMCID: PMC3633456.
63. Garcia-Falgueras C, Lagunas N, Calmarza-Font I, Azcoitia I, Diz-Chaves Y, Garcia-Segura LM, Baquedano E, Frago LM, Argente J, Chowen JA. Gender differences in the long-term effects of chronic prenatal stress on the HPA axis and hypothalamic structure in rats. Psychoneuroendocrinology. 2010 Nov;35(10):1525-35. doi: 10.1016/j.psyneuen.2010.05.006. Epub 2010 Jun 16. PubMed PMID: 20558007.
64. Gelez H, Poirier S, Facchinetti P, Allers KA, Wayman C, Bernabe J, Alexandre L, Giuliano F. Neuroanatomical distribution of the melanocortin-4 receptors in male and female rodent brain. J Chem Neuroanat. 2010 Dec;40(4):310-24. doi: 10.1016/j.jchemneu.2010.09.002. Epub 2010 Sep 25. PubMed PMID: 20884347.
65. Gentile NE, Andrekanic JD, Karwoski TE, Czambel RK, Rubin RT, Rhodes ME. Sexually diergic hypothalamic-pituitary-adrenal (HPA) responses to single-dose nicotine, continuous nicotine infusion, and nicotine withdrawal by mecamylamine in rats. Brain Res Bull. 2011 May 30;85(3-4):145-52. doi: 10.1016/j.brainresbull.2011.03.001. Epub 2011 Mar 17. PubMed PMID: 21396990;PubMed Central PMCID: PMC3109189.
66. Gomes HL, Graceli JB, Gon√ßalves WL, dos Santos RL, Abreu GR, Bissoli NS, Pires JG, Cicilini MA, Moys√©s MR. Influence of gender and estrous cycle on plasma and renal catecholamine levels in rats. Can J Physiol Pharmacol. 2012 Jan;90(1):75-82. doi: 10.1139/y11-102. Epub 2012 Jan 4. PubMed PMID: 22217235.
67. Gonzalez CR, Novelle MG, Caminos JE, Vszquez MJ, Luque RM, Lopez M, Nogueiras R, Di√©guez C. Regulation of lipin1 by nutritional status, adiponectin, sex and pituitary function in rat white adipose tissue. Physiol Behav. 2012 Feb1;105(3):777-83. doi: 10.1016/j.physbeh.2011.10.016. Epub 2011 Oct 25. PubMed PMID: 22051776.
68. Goodarzi S, Pazirandeh A, Jameie SB, Khojasteh NB. Differentiation in boron distribution in adult male and female rats' normal brain: a BNCT approach. Appl Radiat Isot. 2012 Jun;70(6):952-6. doi: 10.1016/j.apradiso.2012.03.021. Epub 2012Mar 30. PubMed PMID: 22484141.
69. Gottschalk S, Cummins CL, Leibfritz D, Christians U, Benet LZ, Serkova NJ. Age and sex differences in the effects of the immunosuppressants cyclosporine, sirolimus and everolimus on rat brain metabolism. Neurotoxicology. 2011 Jan;32(1):50-7. doi: 10.1016/j.neuro.2010.10.006. Epub 2010 Nov 12. PubMed PMID: 21075140.
70. Grassi S, Frondaroli A, Scarduzio M, Dieni CV, Brecchia G, Boiti C, Pettorossi VE. Influence of sex and estrous cycle on synaptic responses of the medial vestibular nuclei in rats: role of circulating 17Œ≤-estradiol. Brain Res Bull. 2012 Feb 10;87(2-3):319-27. doi: 10.1016/j.brainresbull.2011.11.008. Epub2011 Nov 23. PubMed PMID: 22127323.
71. Gray C, Al-Dujaili EA, Sparrow AJ, Gardiner SM, Craigon J, Welham SJ, Gardner DS. Excess maternal salt intake produces sex-specific hypertension in offspring: putative roles for kidney and gastrointestinal sodium handling. PLoS One. 2013 Aug 22;8(8):e72682. doi: 10.1371/journal.pone.0072682. eCollection 2013. PubMed PMID: 23991143; PubMed Central PMCID: PMC3749995.
72. Greco R, Tassorelli C, Mangione AS, Smeraldi A, Allena M, Sandrini G, Nappi G, Nappi RE. Effect of sex and estrogens on neuronal activation in an animal model of migraine. Headache. 2013 Feb;53(2):288-96. doi: 10.1111/j.1526-4610.2012.02249.x. Epub 2012 Aug 22. PubMed PMID: 22913654.
73. Guevara R, Gianotti M, Oliver J, Roca P. Age and sex-related changes in rat brain mitochondrial oxidative status. Exp Gerontol. 2011 Nov;46(11):923-8. doi:10.1016/j.exger.2011.08.003. Epub 2011 Aug 12. PubMed PMID: 21864669.
74. Guevara R, Gianotti M, Roca P, Oliver J. Age and sex-related changes in rat brain mitochondrial function. Cell Physiol Biochem. 2011;27(3-4):201-6. doi:10.1159/000327945. Epub 2011 Apr 1. PubMed PMID: 21471708.
75. Hagiwara H, Funabashi T, Akema T, Kimura F. Sex-specific differences in pain response by dopamine in the bed nucleus of the stria terminalis in rats. Neuroreport. 2013 Mar 6;24(4):181-5. doi: 10.1097/WNR.0b013e32835d8540. PubMed PMID: 23348592.
76. Hajali V, Sheibani V, Esmaeili-Mahani S, Shabani M. Female rats are more susceptible to the deleterious effects of paradoxical sleep deprivation on cognitive performance. Behav Brain Res. 2012 Mar 17;228(2):311-8. doi:10.1016/j.bbr.2011.12.008. Epub 2011 Dec 14. PubMed PMID: 22192378.
77. Hawley WR, Grissom EM, Barratt HE, Conrad TS, Dohanich GP. The effects of biological sex and gonadal hormones on learning strategy in adult rats. Physiol Behav. 2012 Feb 28;105(4):1014-20. doi: 10.1016/j.physbeh.2011.11.021. Epub 2011 Nov 29. PubMed PMID: 22146478.
78. He Z, Ferguson SA, Cui L, Greenfield LJ Jr, Paule MG. Role of neural stem cell activity in postweaning development of the sexually dimorphic nucleus of the preoptic area in rats. PLoS One. 2013;8(1):e54927. doi:10.1371/journal.pone.0054927. Epub 2013 Jan 30. PubMed PMID: 23383001; PubMedCentral PMCID: PMC3559780.
79. Heinzlmann A, Koves K, Kovacs M, Csernus V. Sexual dimorphism in the effect of concomitant progesterone administration on changes caused by long-term estrogen treatment in pituitary hormone immunoreactivities of rats. Med Sci Monit. 2011 Feb 25;17(3):BR62-73. PubMed PMID: 21358595; PubMed Central PMCID: PMC3524720.
80. Heydenreich N, Nolte MW, G√∂b E, Langhauser F, Hofmeister M, Kraft P, Albert-Weissenberger C, Brede M, Varallyay C, G√∂bel K, Meuth SG, Nieswandt B, Dickneite G, Stoll G, Kleinschnitz C. C1-inhibitor protects from brain ischemia-reperfusion injury by combined antiinflammatory and antithrombotic mechanisms. Stroke. 2012 Sep;43(9):2457-67. doi: 10.1161/STROKEAHA.112.660340. Epub 2012 Jun 28. PubMed PMID: 22744646.
81. Hill CA, Alexander ML, McCullough LD, Fitch RH. Inhibition of X-linked inhibitor of apoptosis with embelin differentially affects male versus female behavioral outcome following neonatal hypoxia-ischemia in rats. Dev Neurosci. 2011;33(6):494-504. doi: 10.1159/000331651. Epub 2011 Oct 27. PubMed PMID:22041713; PubMed Central PMCID: PMC3357172.
82. Hillerer KM, Neumann ID, Couillard-Despres S, Aigner L, Slattery DA. Sex-dependent regulation of hippocampal neurogenesis under basal and chronic stress conditions in rats. Hippocampus. 2013 Jun;23(6):476-87. doi:10.1002/hipo.22107. Epub 2013 Mar 18. PubMed PMID: 23504963.
83. Holly EN, Shimamoto A, Debold JF, Miczek KA. Sex differences in behavioral and neural cross-sensitization and escalated cocaine taking as a result of episodic social defeat stress in rats. Psychopharmacology (Berl). 2012 Nov;224(1):179-88. doi: 10.1007/s00213-012-2846-2. Epub 2012 Aug 25. PubMed PMID:22926005; PubMed Central PMCID: PMC3684960.
84. Hosseini M, Sadeghnia HR, Salehabadi S, Soukhtanloo M. Contribution of estradiol in sex-dependent differences of pentylenetetrazole-induced seizures in rats. Acta Physiol Hung. 2013 Jun;100(2):237-45. doi:10.1556/APhysiol.100.2013.004. PubMed PMID: 23524184.
85. Huang CX, Qiu X, Wang S, Wu H, Xia L, Li C, Gao Y, Zhang L, Xiu Y, Chao F, Tang Y. Exercise-induced changes of the capillaries in the cortex of middle-aged rats. Neuroscience. 2013 Mar 13;233:139-45. doi:10.1016/j.neuroscience.2012.12.046. Epub 2013 Jan 3. PubMed PMID: 23291455.
86. Huang Y, Chen S, Xu H, Yu X, Lai H, Ho G, Huang Q, Shi X. Pre-gestational stress alters stress-response of pubertal offspring rat in sexually dimorphic and hemispherically asymmetric manner. BMC Neurosci. 2013 Jul 8;14:67. doi:10.1186/1471-2202-14-67. PubMed PMID: 23829597; PubMed Central PMCID: PMC3707759.
87. Hulshof HJ, Novati A, Luiten PG, den Boer JA, Meerlo P. Despite higher glucocorticoid levels and stress responses in female rats, both sexes exhibit similar stress-induced changes in hippocampal neurogenesis. Behav Brain Res. 2012 Oct 1;234(2):357-64. doi: 10.1016/j.bbr.2012.07.011. Epub 2012 Jul 16. PubMed PMID: 22814114.
88. Huynh TN, Krigbaum AM, Hanna JJ, Conrad CD. Sex differences and phase of light cycle modify chronic stress effects on anxiety and depressive-like behavior. Behav Brain Res. 2011 Sep 12;222(1):212-22. doi: 10.1016/j.bbr.2011.03.038. Epub 2011 Mar 31. PubMed PMID: 21440009.
89. Irwin LN, Byers DM. Novel odors affect gene expression for cytokines and proteinases in the rat amygdala and hippocampus. Brain Res. 2012 Dec 13;1489:1-7. doi: 10.1016/j.brainres.2012.10.034. Epub 2012 Oct 26. PubMed PMID: 23103411; PubMed Central PMCID: PMC3501538.
90. Ishii N, Toda K, Kawakami S, Morozumi M, Yamada T. Receptive field characteristics of stretch-insensitive mechanosensitive units in the rat urinary bladder. Auton Neurosci. 2012 Nov 2;171(1-2):8-13. doi:10.1016/j.autneu.2012.08.004. Epub 2012 Sep 14. PubMed PMID: 22981188.
91. Jacobson-Pick S, Richter-Levin G. Differential impact of juvenile stress and corticosterone in juvenility and in adulthood, in male and female rats. Behav Brain Res. 2010 Dec 25;214(2):268-76. doi: 10.1016/j.bbr.2010.05.036. Epub 2010 Jun 1. PubMed PMID: 20561965.
92. Ji Y, Tang B, Cao DY, Wang G, Traub RJ. Sex differences in spinal processing of transient and inflammatory colorectal stimuli in the rat. Pain. 2012 Sep;153(9):1965-73. doi: 10.1016/j.pain.2012.06.019. Epub 2012 Jul 20. PubMed PMID: 22819535; PubMed Central PMCID: PMC3413769.
93. Johnson RT, Schneider A, DonCarlos LL, Breedlove SM, Jordan CL. Astrocytes in the rat medial amygdala are responsive to adult androgens. J Comp Neurol. 2012 Aug 1;520(11):2531-44. doi: 10.1002/cne.23061. PubMed PMID: 22581688.
94. Jones BA, Watson NV. Perinatal BPA exposure demasculinizes males in measures of affect but has no effect on water maze learning in adulthood. Horm Behav. 2012 Apr;61(4):605-10. doi: 10.1016/j.yhbeh.2012.02.011. Epub 2012 Feb 17. PubMed PMID: 22370244.
95. Keeley RJ, Tyndall AV, Scott GA, Saucier DM. Sex difference in cue strategy in a modified version of the Morris water task: correlations between brain and behaviour. PLoS One. 2013 Jul 17;8(7):e69727. doi: 10.1371/journal.pone.0069727. Print 2013. PubMed PMID: 23874990; PubMed Central PMCID: PMC3714246.
96. Kerstetter KA, Ballis MA, Duffin-Lutgen S, Carr AE, Behrens AM, Kippin TE. Sex differences in selecting between food and cocaine reinforcement are mediated by estrogen. Neuropsychopharmacology. 2012 Nov;37(12):2605-14. doi:10.1038/npp.2012.99. Epub 2012 Aug 8. PubMed PMID: 22871910; PubMed Central PMCID: PMC3473343.
97. Keser A, Balkan B, Gozen O, Kanit L, Pogun S. Hippocampal neuronal nitric oxide synthase (nNOS) is regulated by nicotine and stress in female but not in male rats. Brain Res. 2011 Jan 12;1368:134-42. doi: 10.1016/j.brainres.2010.10.090. Epub 2010 Nov 2. PubMed PMID: 21050840.
98. Kitson AP, Smith TL, Marks KA, Stark KD. Tissue-specific sex differences in docosahexaenoic acid and Œî6-desaturase in rats fed a standard chow diet. Appl Physiol Nutr Metab. 2012 Dec;37(6):1200-11. doi: 10.1139/h2012-103. Epub 2012 Oct 10. PubMed PMID: 23050796.
99. Klug M, van den Buuse M. Chronic cannabinoid treatment during young adulthood induces sex-specific behavioural deficits in maternally separated rats. Behav Brain Res. 2012 Aug 1;233(2):305-13. doi: 10.1016/j.bbr.2012.05.019. Epub2012 May 17. PubMed PMID: 22610052.
100. Kokras N, Sotiropoulos I, Pitychoutis PM, Almeida OF, Papadopoulou-Daifoti, Z. Citalopram-mediated anxiolysis and differing neurobiological responses in both sexes of a genetic model of depression. Neuroscience. 2011 Oct 27;194:62-71. doi: 10.1016/j.neuroscience.2011.07.077. Epub 2011 Aug 4. PubMed PMID: 21839808.
101. Konkle AT, McCarthy MM. Developmental time course of estradiol, testosterone, and dihydrotestosterone levels in discrete regions of male and female rat brain. Endocrinology. 2011 Jan;152(1):223-35. doi:10.1210/en.2010-0607. Epub 2010 Nov 10. PubMed PMID: 21068160; PubMed CentralPMCID: PMC3033055.
102. Koss WA, Franklin AD, Juraska JM. Delayed alternation in adolescent and adult male and female rats. Dev Psychobiol. 2011 Nov;53(7):724-31. doi: 0.1002/dev.20543. Epub 2011 Mar 22. PubMed PMID: 21432847.
103. Koyama M, Yin C, Ishii H, Sakuma Y, Kato M. Somatostatin inhibition of GnRH neuronal activity and the morphological relationship between GnRH and somatostatin neurons in rats. Endocrinology. 2012 Feb;153(2):806-14. doi:10.1210/en.2011-1374. Epub 2011 Dec 6. PubMed PMID: 22147011.
104. Kucka M, Bjelobaba I, Clokie SJ, Klein DC, Stojilkovic SS. Female-specific induction of rat pituitary dentin matrix protein-1 by GnRH. Mol Endocrinol. 2013 Nov;27(11):1840-55. doi: 10.1210/me.2013-1068. Epub 2013 Oct 1. PubMed PMID: 24085820; PubMed Central PMCID: PMC3805844.
105. Lacy RT, Mactutus CF, Harrod SB. Prenatal IV nicotine exposure produces a sex difference in sensorimotor gating of the auditory startle reflex in adult rats. Int J Dev Neurosci. 2011 Apr;29(2):153-61. doi: 10.1016/j.ijdevneu.2010.12.001. Epub 2010 Dec 8. PubMed PMID: 21145386; PubMed Central PMCID: PMC3312379.
106. Lafuente A, Pereiro N. Neurotoxic effects induced by endosulfan exposure during pregnancy and lactation in female and male rat striatum. Toxicology. 2013 Sep 6;311(1-2):35-40. doi: 10.1016/j.tox.2013.05.001. Epub 2013 May 20. PubMed PMID: 23702353.
107. Laplante F, Brake WG, Chehab SL, Sullivan RM. Sex differences in the effects of perinatal anoxia on dopamine function in rats. Neurosci Lett. 2012 Jan 6;506(1):89-93. doi: 10.1016/j.neulet.2011.10.055. Epub 2011 Oct 30. PubMed PMID: 22061835.
108. Lariviere WR, Fiorenzani P, Ceccarelli I, Massafra C, Sorda G, Di Canio C, Aloisi AM. Central CRH administration changes formalin pain responses in male and female rats. Brain Res. 2011 Apr 6;1383:128-34. doi: 10.1016/j.brainres.2011.01.106. Epub 2011 Feb 25. PubMed PMID: 21300038.
109. Lasley SM, Gilbert ME. Developmental thyroid hormone insufficiency reduces expression of brain-derived neurotrophic factor (BDNF) in adults but not in neonates. Neurotoxicol Teratol. 2011 Jul-Aug;33(4):464-72. doi: 10.1016/j.ntt.2011.04.001. Epub 2011 Apr 17. PubMed PMID: 21530650.
110. Lebron-Milad K, Tsareva A, Ahmed N, Milad MR. Sex differences and estrous cycle in female rats interact with the effects of fluoxetine treatment on fear extinction. Behav Brain Res. 2013 Sep 15;253:217-22. doi: 10.1016/j.bbr.2013.07.024. Epub 2013 Jul 22. PubMed PMID: 23886596; PubMed Central PMCID: PMC4106477.
111. Lenglos C, Mitra A, Gu√®vremont G, Timofeeva E. Sex differences in the effects of chronic stress and food restriction on body weight gain and brain expression of CRF and relaxin-3 in rats. Genes Brain Behav. 2013 Jun;12(4):370-87. doi: 10.1111/gbb.12028. Epub 2013 Mar 12. PubMed PMID: 23425370.
112. Li JN, Qian Z, Xu WX, Xu B, Lu XL, Yan ZY, Han LM, Liu Y, Yuan M, Schild J, Qiao GF, Li BY. Gender differences in histamine-induced depolarization and inward currents in vagal ganglion neurons in rats. Int J Biol Sci. 2013 Nov 20;9(10):1079-88. doi: 10.7150/ijbs.7595. eCollection 2013. PubMed PMID: 24339729; PubMed Central PMCID: PMC3858581.
113. Little JM, Qin C, Farber JP, Foreman RD. Spinal cord processing of cardiacnociception: are there sex differences between male and proestrous female rats? Brain Res. 2011 Sep 21;1413:24-31. doi: 10.1016/j.brainres.2011.07.036. Epub 2011 Jul 23. PubMed PMID: 21839425; PubMed Central PMCID: PMC3167014.
114. Liu NJ, Schnell S, Wessendorf MW, Gintzler AR. Sex, pain, and opioids: interdependent influences of sex and pain modality on dynorphin-mediated antinociception in rats. J Pharmacol Exp Ther. 2013 Feb;344(2):522-30. doi: 10.1124/jpet.112.199851. Epub 2012 Dec 10. PubMed PMID: 23230215.
115. 10.1016/j.neuroscience.2011.11.033. Epub 2011 Nov 22. PubMed PMID: 22120435.
116. Llorente-Berzal A, Assis MA, Rubino T, Zamberletti E, Marco EM, Parolaro D, Ambrosio E, Viveros MP. Sex-dependent changes in brain CB1R expression and functionality and immune CB2R expression as a consequence of maternal deprivation and adolescent cocaine exposure. Pharmacol Res. 2013 Aug;74:23-33. doi: 10.1016/j.phrs.2013.05.001. Epub 2013 May 14. PubMed PMID: 23680694.
117. Logrip ML, Rivier C, Lau C, Im S, Vaughan J, Lee S. Adolescent alcohol exposure alters the rat adult hypothalamic-pituitary-adrenal axis responsiveness in a sex-specific manner. Neuroscience. 2013 Apr 3;235:174-86. doi:10.1016/j.neuroscience.2012.12.069. Epub 2013 Jan 18. PubMed PMID: 23337533;PubMed Central PMCID: PMC3595399.
118. Luo H, Deng Z, Liu L, Shen L, Kou H, He Z, Ping J, Xu D, Ma L, Chen L, Wang H. Prenatal caffeine ingestion induces transgenerational neuroendocrine metabolic programming alteration in second generation rats. Toxicol Appl Pharmacol. 2014 Feb 1;274(3):383-92. doi: 10.1016/j.taap.2013.11.020. Epub 2013 Dec 8. PubMed PMID: 24321341.
119. Luo J, Wang T, Liang S, Hu X, Li W, Jin F. Experimental gastritis leads to anxiety- and depression-like behaviors in female but not male rats. Behav Brain Funct. 2013 Dec 17;9:46. doi: 10.1186/1744-9081-9-46. PubMed PMID: 24345032; PubMed Central PMCID: PMC3878489.
120. Lopez-Aumatell R, Martinez-Membrives E, Vicens-Costa E, Ca√±ete T, Bl√°zquez G, Mont-Cardona C, Johannesson M, Flint J, Tobe√±a A, Fern√°ndez-Teruel A. Effects of environmental and physiological covariates on sex differences in unconditioned and conditioned anxiety and fear in a large sample of genetically heterogeneous (N/Nih-HS) rats. Behav Brain Funct. 2011 Nov 25;7:48. doi: 10.1186/1744-9081-7-48. PubMed PMID: 22118015; PubMed Central PMCID: PMC3254066.
121. Lopez-Gallardo M, Lopez-Rodriguez AB, Llorente-Berzal Å, Rotllant D, Mackie K, Armario A, Nadal R Viveros MP. Maternal deprivation and adolescent cannabinoid exposure impact hippocampal astrocytes, CB1 receptors and brain-derived neurotrophic factor in a sexually dimorphic fashion. Neuroscience. 2012 Mar 1;204:90-103. doi: 10.1016/j.neuroscience.2011.09.063. Epub 2011 Oct 6. PubMed PMID: 22001306; PubMed Central PMCID: PMC3659815.
122. Ma Y, Sullivan JC, Schreihofer DA. Dietary genistein and equol (4', 7 isoflavandiol) reduce oxidative stress and protect rats against focal cerebral ischemia. Am J Physiol Regul Integr Comp Physiol. 2010 Sep;299(3):R871-7. doi: 10.1152/ajpregu.00031.2010. Epub 2010 Jul 14. PubMed PMID: 20631292.
123. impair learning after acute stress in females but not in males. J Neurosci. 2010 Dec 1;30(48):16188-96. doi: 10.1523/JNEUROSCI.2265-10.2010. PubMed PMID: 21123565; PubMed Central PMCID: PMC3073607.
124. Mansouri MT, Naghizadeh B, L√≥pez-Larrubia P, Cauli O. Gender-dependent behavioural impairment and brain metabolites in young adult rats after short term exposure to lead acetate. Toxicol Lett. 2012 Apr 5;210(1):15-23. doi:10.1016/j.toxlet.2012.01.012. Epub 2012 Jan 25. PubMed PMID: 22285975.
125. Mansouri MT, Naghizadeh B, L√≥pez-Larrubia P, Cauli O. Behavioral deficits induced by lead exposure are accompanied by serotonergic and cholinergic alterations in the prefrontal cortex. Neurochem Int. 2013 Feb;62(3):232-9. doi: 10.1016/j.neuint.2012.12.009. Epub 2012 Dec 22. PubMed PMID: 23266395.
126. Maris AF, Franco JL, Mitozo PA, Paviani G, Borowski C, Trevisan R, Uliano-Silva M, Farina M, Dafre AL. Gender effects of acute malathion or zinc exposure on the antioxidant response of rat hippocampus and cerebral cortex. Basic Clin Pharmacol Toxicol. 2010 Dec;107(6):965-70. doi: 10.1111/j.1742-7843.2010.00614.x. PubMed PMID: 20629655.
127. Markham JA, Mullins SE, Koenig JI. Periadolescent maturation of the prefrontal cortex is sex-specific and is disrupted by prenatal stress. J Comp Neurol. 2013 Jun 1;521(8):1828-43. doi: 10.1002/cne.23262. PubMed PMID: 23172080.
128. Massella A, D'Intino G, Fern√°ndez M, Sivilia S, Lorenzini L, Giatti S, Melcangi RC, Calz√† L, Giardino L. Gender effect on neurodegeneration and myelin markers in an animal model for multiple sclerosis. BMC Neurosci. 2012 Jan 24;13:12. doi: 10.1186/1471-2202-13-12. PubMed PMID: 22272832; PubMed Central PMCID: PMC3282645.
129. Matsuda KI, Mori H, Nugent BM, Pfaff DW, McCarthy MM, Kawata M. Histone deacetylation during brain development is essential for permanent masculinization of sexual behavior. Endocrinology. 2011 Jul;152(7):2760-7. doi: 10.1210/en.2011-0193. Epub 2011 May 17. PubMed PMID: 21586557; PubMed Central PMCID: PMC3115610.
130. Mehta NS, Wang L, Redei EE. Sex differences in depressive, anxious behaviors and hippocampal transcript levels in a genetic rat model. Genes Brain Behav. 2013 Oct;12(7):695-704. doi: 10.1111/gbb.12063. Epub 2013 Aug 6. PubMed PMID: 23876038.
131. Meitzen J, Perry AN, Westenbroek C, Hedges VL, Becker JB, Mermelstein PG. Enhanced striatal Œ≤1-adrenergic receptor expression following hormone loss in adulthood is programmed by both early sexual differentiation and puberty: a study of humans and rats. Endocrinology. 2013 May;154(5):1820-31. doi: 10.1210/en.2012-2131. Epub 2013 Mar 26. PubMed PMID: 23533220; PubMed Central PMCID: PMC3628022.
132. Mierzejewska-Krzy≈ºowska B, Drzyma≈Ça-Celichowska H, Celichowski J. Gender differences in the morphometric properties of muscle fibres and the innervation ratio of motor units in rat medial gastrocnemius muscle. Anat Histol Embryol. 2011 Aug;40(4):249-55. doi: 10.1111/j.1439-0264.2011.01066.x. Epub 2011 Mar 22. PubMed PMID: 21426379.
133. Mikroulis AV, Psarropoulou C. Endogenous ACh effects on NMDA-inducedinterictal-like discharges along the septotemporal hippocampal axis of adult rats and their modulation by an early life generalized seizure. Epilepsia. 2012 May;53(5):879-87. doi: 10.1111/j.1528-1167.2012.03440.x. Epub 2012 Mar 16. PubMed PMID: 22428538.
134. Milner TA, Burstein SR, Marrone GF, Khalid S, Gonzalez AD, Williams TJ, Schierberl KC, Torres-Reveron A, Gonzales KL, McEwen BS, Waters EM. Stress differentially alters mu opioid receptor density and trafficking in parvalbumin-containing interneurons in the female and male rat hippocampus. Synapse. 2013 Nov;67(11):757-72. doi: 10.1002/syn.21683. Epub 2013 Jul 17. PubMed PMID: 23720407; PubMed Central PMCID: PMC3778032.
135. Mitic M, Simic I, Djordjevic J, Radojcic MB, Adzic M. Gender-specific effects of fluoxetine on hippocampal glucocorticoid receptor phosphorylation and behavior in chronically stressed rats. Neuropharmacology. 2013 Jul;70:100-11.doi: 10.1016/j.neuropharm.2012.12.012. Epub 2013 Jan 23. PubMed PMID: 23353902.
136. Mohagheghi F, Khalaj L, Ahmadiani A, Rahmani B. Gemfibrozil pretreatment affecting antioxidant defense system and inflammatory, but not Nrf-2 signaling pathways resulted in female neuroprotection and male neurotoxicity in the rat models of global cerebral ischemia-reperfusion. Neurotox Res. 2013 Apr;23(3):225-37. doi: 10.1007/s12640-012-9338-3. Epub 2012 Jul 7. PubMed PMID: 22773136.
137. Moreira AC, Silva AM, Santos MS, Sard√£o VA. Resveratrol affects differently rat liver and brain mitochondrial bioenergetics and oxidative stress in vitro: investigation of the role of gender. Food Chem Toxicol. 2013 Mar;53:18-26. doi: 10.1016/j.fct.2012.11.031. Epub 2012 Nov 29. PubMed PMID: 23200887.
138. Mourlon V, Baudin A, Blanc O, Lauber A, Giros B, Naudon L, Daug√© V. Maternal deprivation induces depressive-like behaviours only in female rats. Behav Brain Res. 2010 Dec 1;213(2):278-87. doi: 10.1016/j.bbr.2010.05.017. Epub 2010 May 19. PubMed PMID: 20488211.
139. Mourlon V, Naudon L, Giros B, Crumeyrolle-Arias M, Daug√© V. Early stress leads to effects on estrous cycle and differential responses to stress. Physiol Behav. 2011 Mar 1;102(3-4):304-10. doi: 10.1016/j.physbeh.2010.11.003. Epub 2010 Nov 6. PubMed PMID: 21059366.
140. Muhammad A, Hossain S, Pellis SM, Kolb B. Tactile stimulation during development attenuates amphetamine sensitization and structurally reorganizes prefrontal cortex and striatum in a sex-dependent manner. Behav Neurosci. 2011 Apr;125(2):161-74. doi: 10.1037/a0022628. PubMed PMID: 21463020.
141. Muneoka K, Kuwagata M, Ogawa T, Shioda S. Sex-specific effects of early neonatal progesterone treatment on dopamine and serotonin metabolism in rat striatum and frontal cortex. Life Sci. 2010 Dec 18;87(23-26):738-42. doi: 10.1016/j.lfs.2010.10.016. Epub 2010 Oct 27. PubMed PMID: 21034751.
142. Nasiraei-Moghadam S, Sherafat MA, Safari MS, Moradi F, Ahmadiani A, Dargahi L. Reversal of prenatal morphine exposure-induced memory deficit in male but not female rats. J Mol Neurosci. 2013 May;50(1):58-69. doi:10.1007/s12031-012-9860-z. Epub 2012 Aug 3. PubMed PMID: 22864979.
143. Nesil T, Kanit L, Collins AC, Pogun S. Individual differences in oral nicotine intake in rats. Neuropharmacology. 2011 Jul-Aug;61(1-2):189-201. doi: 10.1016/j.neuropharm.2011.03.027. Epub 2011 Apr 12. PubMed PMID: 21504750; PubMed Central PMCID: PMC3105211.
144. Niu KY, Zhang Y, Ro JY. Effects of gonadal hormones on the peripheral cannabinoid receptor 1 (CB1R) system under a myositis condition in rats. Pain. 2012 Nov;153(11):2283-91. doi: 10.1016/j.pain.2012.07.037. Epub 2012 Aug 31. PubMed PMID: 22940464; PubMed Central PMCID: PMC3578305.
145. Nogami H, Lee Mc, Soya H, Hisano S. Regional distribution and ontogeny of the first exon variants of the rat growth hormone receptor mRNA in the brain and the pituitary gland. Growth Horm IGF Res. 2011 Feb;21(1):11-5. doi: 10.1016/j.ghir.2010.11.001. Epub 2010 Dec 22. PubMed PMID: 21177131.
146. Noschang C, Krolow R, Arcego DM, Toniazzo AP, Huffell AP, Dalmaz C. Neonatal handling affects learning, reversal learning and antioxidant enzymes activities in a sex-specific manner in rats. Int J Dev Neurosci. 2012 Jun;30(4):285-91. doi:10.1016/j.ijdevneu.2012.01.010. Epub 2012 Feb 2. PubMed PMID: 22326443.
147. Nygard SK, Klambatsen A, Hazim R, Eltareb MH, Blank JC, Chang AJ, Quinones-Jenab V, Jenab S. Sexually dimorphic intracellular responses after cocaine-induced conditioned place preference expression. Brain Res. 2013 Jul 3;1520:121-33. doi: 10.1016/j.brainres.2013.04.060. Epub 2013 May 9. PubMed PMID: 23665060; PubMed Central PMCID: PMC3964786.
148. Noslund J, Studer E, Nilsson K, Westberg L, Eriksson E. Serotonin depletion counteracts sex differences in anxiety-related behaviour in rat. Psychopharmacology (Berl). 2013 Nov;230(1):29-35. doi: 10.1007/s00213-013-3133-6. Epub 2013 May 17. PubMed PMID: 23681161.
149. Olczak M, Duszczyk M, Mierzejewski P, Meyza K, Majewska MD. Persistent behavioral impairments and alterations of brain dopamine system after early postnatal administration of thimerosal in rats. Behav Brain Res. 2011 Sep 30;223(1):107-18. doi: 10.1016/j.bbr.2011.04.026. Epub 2011 Apr 28. PubMed PMID: 21549155.
150. Olea E, Gaytan SP, Obeso A, Gonzalez C, Pasaro R. Interactions between postnatal sustained hypoxia and intermittent hypoxia in the adulthood to alter brainstem structures and respiratory function. Adv Exp Med Biol. 2012;758:225-31. doi: 10.1007/978-94-007-4584-1_31. PubMed PMID: 23080166.
151. Ong ZY, Wanasuria AF, Lin MZ, Hiscock J, Muhlhausler BS. Chronic intake of a cafeteria diet and subsequent abstinence. Sex-specific effects on gene expression in the mesolimbic reward system. Appetite. 2013 Jun;65:189-99. doi:10.1016/j.appet.2013.01.014. Epub 2013 Feb 10. PubMed PMID: 23402719.
152. Overgaard A, Tena-Sempere M, Franceschini I, Desroziers E, Simonneaux V, Mikkelsen JD. Comparative analysis of kisspeptin-immunoreactivity reveals genuine differences in the hypothalamic Kiss1 systems between rats and mice. Peptides. 2013 Jul;45:85-90. doi: 10.1016/j.peptides.2013.04.013. Epub 2013 May 4. PubMed PMID: 23651990.
153. Patel DA, Booze RM, Mactutus CF. Prenatal cocaine exposure alters progenitor cell markers in the subventricular zone of the adult rat brain. Int J Dev Neurosci. 2012 Feb;30(1):1-9. doi: 10.1016/j.ijdevneu.2011.11.001. Epub 2011 Nov 17. PubMed PMID: 22119286; PubMed Central PMCID: PMC3825177.
154. Patil MJ, Ruparel SB, Henry MA, Akopian AN. Prolactin regulates TRPV1, TRPA1, and TRPM8 in sensory neurons in a sex-dependent manner: Contribution of prolactin receptor to inflammatory pain. Am J Physiol Endocrinol Metab. 2013 Nov 1;305(9):E1154-64. doi: 10.1152/ajpendo.00187.2013. Epub 2013 Sep 10. PubMedPMID: 24022869; PubMed Central PMCID: PMC3840203.
155. Patten AR, Sickmann HM, Dyer RA, Innis SM, Christie BR. Omega-3 fatty acids can reverse the long-term deficits in hippocampal synaptic plasticity caused by prenatal ethanol exposure. Neurosci Lett. 2013 Sep 13;551:7-11. doi:10.1016/j.neulet.2013.05.051. Epub 2013 Jul 18. PubMed PMID: 23872044.
156. Pereno GL, Balaszczuk V, Beltramino CA. Effect of sex differences and gonadal hormones on kainic acid-induced neurodegeneration in the bed nucleus of the stria terminalis of the rat. Exp Toxicol Pathol. 2012 May;64(4):283-9. doi:10.1016/j.etp.2010.08.020. Epub 2010 Sep 28. PubMed PMID: 20880685.
157. Pereno GL, Balaszczuk V, Beltramino CA. Detection of conspecific pheromones elicits fos expression in GABA and calcium-binding cells of the rat vomeronasal system-medial extended amygdala. J Physiol Biochem. 2011 Mar;67(1):71-85. doi: 10.1007/s13105-010-0051-5. Epub 2010 Oct 12. PubMed PMID: 20938761.
158. Perry AN, Westenbroek C, Becker JB. The development of a preference for cocaine over food identifies individual rats with addiction-like behaviors. PLoS One. 2013 Nov 18;8(11):e79465. doi: 10.1371/journal.pone.0079465. eCollection 2013. PubMed PMID: 24260227; PubMed Central PMCID: PMC3832528.
159. Pesaresi M, Giatti S, Cavaletti G, Abbiati F, Calabrese D, Lombardi R, Bianchi R, Lauria G, Caruso D, Garcia-Segura LM, Melcangi RC. Sex-dimorphic effects of dehydroepiandrosterone in diabetic neuropathy. Neuroscience. 2011 Dec 29;199:401-9. doi: 10.1016/j.neuroscience.2011.09.013. Epub 2011 Sep 16. PubMedPMID: 21945035.
160. Pesaresi M, Giatti S, Cavaletti G, Abbiati F, Calabrese D, Bianchi R, Caruso D, Garcia-Segura LM, Melcangi RC. Sex differences in the manifestation of peripheral diabetic neuropathy in gonadectomized rats: a correlation with the levels of neuroactive steroids in the sciatic nerve. Exp Neurol. 2011 Apr;228(2):215-21. doi: 10.1016/j.expneurol.2011.01.005. Epub 2011 Jan 15. PubMed PMID: 21241692.
161. Petrovich GD, Lougee MA. Sex differences in fear-induced feeding cessation: prolonged effect in female rats. Physiol Behav. 2011 Oct 24;104(5):996-1001. doi: 10.1016/j.physbeh.2011.06.020. Epub 2011 Jul 1. PubMed PMID: 21745485.
162. Pinheiro CR, Oliveira E, Trevenzoli IH, Manh√£es AC, Santos-Silva AP, Younes-Rapozo V, Claudio-Neto S, Santana AC, Nascimento-Saba CC, Moura EG, Lisboa PC. Developmental plasticity in adrenal function and leptin production primed by nicotine exposure during lactation: gender differences in rats. Horm Metab Res. 2011 Sep;43(10):693-701. doi: 10.1055/s-0031-1285909. Epub 2011 Sep 19. PubMed PMID: 21932173.
163. Pinkham MI, Guild SJ, Malpas SC, Barrett CJ. Effects of sex and ovarian hormones on the initial renal sympathetic nerve activity response to myocardial infarction. Exp Physiol. 2012 Sep;97(9):1040-53. doi: 10.1113/expphysiol.2012.065615. Epub 2012 May 4. PubMed PMID: 22562810.
164. Pinos H, Perez-Izquierdo MA, Carrillo B, Collado P. Effects of undernourishment on the hypothalamic orexinergic system. Physiol Behav. 2011 Jan 10;102(1):17-21. doi: 10.1016/j.physbeh.2010.09.023. Epub 2010 Oct 13. PubMed PMID: 20932853.
165. Piontkewitz Y, Arad M, Weiner I. Abnormal trajectories of neurodevelopment and behavior following in utero insult in the rat. Biol Psychiatry. 2011 Nov1;70(9):842-51. doi: 10.1016/j.biopsych.2011.06.007. Epub 2011 Aug 4. PubMed PMID: 21816387.
166. Pitychoutis PM, Dalla C, Sideris AC, Tsonis PA, Papadopoulou-Daifoti Z. 5-HT(1A), 5-HT(2A), and 5-HT(2C) receptor mRNA modulation by antidepressant treatment in the chronic mild stress model of depression: sex differences exposed. Neuroscience. 2012 May 17;210:152-67. doi: 10.1016/j.neuroscience.2012.03.003. Epub 2012 Mar 9. PubMed PMID: 22441040.
167. Pitychoutis PM, Pallis EG, Mikail HG, Papadopoulou-Daifoti Z. Individual differences in novelty-seeking predict differential responses to chronic antidepressant treatment through sex- and phenotype-dependent neurochemical signatures. Behav Brain Res. 2011 Sep 30;223(1):154-68. doi: 10.1016/j.bbr.2011.04.036. Epub 2011 Apr 27. PubMed PMID: 21549763.
168. Powanda MC, Rainsford KD. A toxicological investigation of a celery seed extract having anti-inflammatory activity. Inflammopharmacology. 2011 Aug;19(4):227-33. doi: 10.1007/s10787-010-0049-1. Epub 2010 Jun 22. PubMed PMID: 20568016.
169. Pyter LM, Kelly SD, Harrell CS, Neigh GN. Sex differences in the effects of adolescent stress on adult brain inflammatory markers in rats. Brain Behav Immun. 2013 May;30:88-94. doi: 10.1016/j.bbi.2013.01.075. Epub 2013 Jan 21. PubMed PMID: 23348027; PubMed Central PMCID: PMC3641183.
170. Pisa A, Kupai K, M√©nesi R, Szalai Z, Szab√≥ R, Pint√©r Z, P√°lfi G, Gy√∂ngy√∂si M, Berk√≥ A, P√°v√≥ I, Varga C. Sexual dimorphism of cardiovascular ischemia susceptibility is mediated by heme oxygenase. Oxid Med Cell Longev. 2013;2013:521563. doi: 10.1155/2013/521563. Epub 2013 Sep 17. PubMed PMID: 24163720; PubMed Central PMCID: PMC3791627.
171. Quintela T, Gon√ßalves I, Carreto LC, Santos MA, Marcelino H, Patriarca FM, Santos CR. Analysis of the effects of sex hormone background on the rat choroid plexus transcriptome by cDNA microarrays. PLoS One. 2013 Apr 9;8(4):e60199. doi: 10.1371/journal.pone.0060199. Print 2013. PubMed PMID: 23585832; PubMed Central PMCID: PMC3622009.
172. Reichel CM, Chan CH, Ghee SM, See RE. Sex differences in escalation of methamphetamine self-administration: cognitive and motivational consequences in rats. Psychopharmacology (Berl). 2012 Oct;223(4):371-80. Epub 2012 May 17. PubMed PMID: 22592902; PubMed Central PMCID: PMC3698560.
173. Ribeiro AM, Barbosa FF, Godinho MR, Fernandes VS, Munguba H, Melo TG, Barbosa MT, Eufrasio RA, Cabral A, Iz√≠dio GS, Silva RH. Sex differences in aversive memory in rats: possible role of extinction and reactive emotional factors. Brain Cogn. 2010 Nov;74(2):145-51. doi: 10.1016/j.bandc.2010.07.012. Epub 2010 Aug 19. PubMed PMID: 20727653.
174. Riebe CJ, Hill MN, Lee TT, Hillard CJ, Gorzalka BB. Estrogenic regulation of limbic cannabinoid receptor binding. Psychoneuroendocrinology. 2010 Sep;35(8):1265-9. doi: 10.1016/j.psyneuen.2010.02.008. Epub 2010 Mar 6. PubMed PMID: 20207489; PubMed Central PMCID: PMC2933663.
175. Rodrigues AR, Ferreira RS, Salgado HC, Fazan VP. Morphometric analysis of the phrenic nerve in male and female Wistar-Kyoto (WKY) and spontaneously hypertensive rats (SHR). Braz J Med Biol Res. 2011 Jun;44(6):583-91. Epub 2011May 2. PubMed PMID: 21537611.
176. Rodriguez CA, Chamizo VD, Mackintosh NJ. Overshadowing and blocking between landmark learning and shape learning: the importance of sex differences. Learn Behav. 2011 Dec;39(4):324-35. doi: 10.3758/s13420-011-0027-5. PubMed PMID:21472414.
177. Ruiz-Pino F, Navarro VM, Bentsen AH, Garcia-Galiano D, Sanchez-Garrido MA, Ciofi P, Steiner RA, Mikkelsen JD, Pinilla L, Tena-Sempere M. Neurokinin B and the control of the gonadotropic axis in the rat: developmental changes, sexual dimorphism, and regulation by gonadal steroids. Endocrinology. 2012 Oct;153(10):4818-29. Epub 2012 Jul 20. PubMed PMID: 22822161; PubMed Central PMCID: PMC3512006.
178. Ryzhavskii BY, Zadvornaya OV, Lebed'ko OA. Effect of treatment with testosterone derivatives on morphometric characteristics and free radical oxidation in rat cerebral cortex. Bull Exp Biol Med. 2012 Oct;153(6):902-6. English, Russian. PubMed PMID: 23113314.
179. Saarinen NM, Thompson LU. Prolonged administration of secoisolariciresinol diglycoside increases lignan excretion and alters lignan tissue distribution in adult male and female rats. Br J Nutr. 2010 Sep;104(6):833-41. doi:10.1017/S0007114510001194. Epub 2010 Apr 14. PubMed PMID: 20388250.
180. Saloman JL, Niu KY, Ro JY. Activation of peripheral delta-opioid receptors leads to anti-hyperalgesic responses in the masseter muscle of male and female rats. Neuroscience. 2011 Sep 8;190:379-85. doi: 10.1016/j.neuroscience.2011.05.062. Epub 2011 Jun 6. PubMed PMID: 21664434; PubMed Central PMCID: PMC3156332.
181. Salomon S, Bejar C, Schorer-Apelbaum D, Weinstock M. Corticosterone mediates some but not other behavioural changes induced by prenatal stress in rats. J Neuroendocrinol. 2011 Feb;23(2):118-28. doi: 10.1111/j.1365-2826.2010.02097.x. PubMed PMID: 21108672.
182. Sanada LS, da Rocha Kalil AL, Tavares MR, Neubern MC, Salgado HC, Fazan VP. Sural nerve involvement in experimental hypertension: morphology and morphometry in male and female normotensive Wistar-Kyoto (WKY) and spontaneously hypertensive rats (SHR). BMC Neurosci. 2012 Mar 2;13:24. doi: 10.1186/1471-2202-13-24. PubMed PMID: 22380617; PubMed Central PMCID: PMC3350456.
183. Sanches EF, Arteni NS, Nicola F, Boisserand L, Willborn S, Netto CA. Early hypoxia-ischemia causes hemisphere and sex-dependent cognitive impairment and histological damage. Neuroscience. 2013 May 1;237:208-15. doi:10.1016/j.neuroscience.2013.01.066. Epub 2013 Feb 6. PubMed PMID: 23395861.
184. Scheff NN, Gold MS. Sex differences in the inflammatory mediator-induced sensitization of dural afferents. J Neurophysiol. 2011 Oct;106(4):1662-8. doi: 10.1152/jn.00196.2011. Epub 2011 Jul 13. PubMed PMID: 21753025; PubMed Central PMCID: PMC3191837.
185. Schwarz JM, Sholar PW, Bilbo SD. Sex differences in microglial colonization of the developing rat brain. J Neurochem. 2012 Mar;120(6):948-63. doi:10.1111/j.1471-4159.2011.07630.x. Epub 2012 Feb 9. PubMed PMID: 22182318; PubMed Central PMCID: PMC3296888.
186. Schwarz JM, Nugent BM, McCarthy MM. Developmental and hormone-induced epigenetic changes to estrogen and progesterone receptor genes in brain are dynamic across the life span. Endocrinology. 2010 Oct;151(10):4871-81. doi: 10.1210/en.2010-0142. Epub 2010 Aug 11. PubMed PMID: 20702577; PubMed CentralPMCID: PMC2946142.
187. Segatto M, Di Giovanni A, Marino M, Pallottini V. Analysis of the protein network of cholesterol homeostasis in different brain regions: an age and sex dependent perspective. J Cell Physiol. 2013 Jul;228(7):1561-7. doi:10.1002/jcp.24315. PubMed PMID: 23280554.
188. Segatto M, Trapani L, Marino M, Pallottini V. Age- and sex-related differences in extra-hepatic low-density lipoprotein receptor. J Cell Physiol. 2011 Oct;226(10):2610-6. doi: 10.1002/jcp.22607. PubMed PMID: 21792919.
189. Shaashua L, Sominsky L, Levi B, Sorski L, Reznick M, Page GG, Ben-Eliyahu S.In vivo suppression of plasma IL-12 levels by acute and chronic stress paradigms: potential mediating mechanisms and sex differences. Brain Behav Immun. 2012 Aug;26(6):996-1005. doi: 10.1016/j.bbi.2012.05.012. Epub 2012 May 29. PubMed PMID: 22659252; PubMed Central PMCID: PMC3398208.
190. Sherrill LK, Berthold C, Koss WA, Juraska JM, Gulley JM. Sex differences in the effects of ethanol pre-exposure during adolescence on ethanol-induced conditioned taste aversion in adult rats. Behav Brain Res. 2011 Nov 20;225(1):104-9. doi: 10.1016/j.bbr.2011.07.003. Epub 2011 Jul 8. PubMed PMID:21767576; PubMed Central PMCID: PMC3170442.
191. Sherrill LK, Koss WA, Foreman ES, Gulley JM. The effects of pre-pubertal gonadectomy and binge-like ethanol exposure during adolescence on ethanol drinking in adult male and female rats. Behav Brain Res. 2011 Jan 20;216(2):569-75. doi: 10.1016/j.bbr.2010.08.048. Epub 2010 Sep 15. PubMed PMID: 20816899; PubMed Central PMCID: PMC2981628.
192. Shneider Y, Shtrauss Y, Yadid G, Pinhasov A. Differential expression of PACAP receptors in postnatal rat brain. Neuropeptides. 2010 Dec;44(6):509-14. doi: 10.1016/j.npep.2010.09.001. Epub 2010 Oct 23. PubMed PMID: 20971507.
193. Silva A, Araujo P, Zager A, Tufik S, Andersen ML. Sex differences in sleep pattern of rats in an experimental model of osteoarthritis. Eur J Pain. 2011 Jul;15(6):545-53. doi: 10.1016/j.ejpain.2010.10.009. Epub 2011 Jan 26. PubMed PMID: 21273100.
194. Singh SP, Kumari M, Kumari SI, Rahman MF, Mahboob M, Grover P. Toxicity assessment of manganese oxide micro and nanoparticles in Wistar rats after 28‚Äâdays of repeated oral exposure. J Appl Toxicol. 2013 Oct;33(10):1165-79. doi: 10.1002/jat.2887. Epub 2013 May 24. PubMed PMID: 23702825.
195. Skwara AJ, Karwoski TE, Czambel RK, Rubin RT, Rhodes ME. Influence of environmental enrichment on hypothalamic-pituitary-adrenal (HPA) responses to single-dose nicotine, continuous nicotine by osmotic mini-pumps, and nicotine withdrawal by mecamylamine in male and female rats. Behav Brain Res. 2012 Sep 1;234(1):1-10. doi: 10.1016/j.bbr.2012.06.003. Epub 2012 Jun 13. PubMed PMID:22705101; PubMed Central PMCID: PMC3417336.
196. Smith AL, Alexander M, Rosenkrantz TS, Sadek ML, Fitch RH. Sex differences in behavioral outcome following neonatal hypoxia ischemia: insights from a clinical meta-analysis and a rodent model of induced hypoxic ischemic brain injury. Exp Neurol. 2014 Apr;254:54-67. doi: 10.1016/j.expneurol.2014.01.003.Epub 2014 Jan 13. PubMed PMID: 24434477.
197. Sterrenburg L, Gaszner B, Boerrigter J, Santbergen L, Bramini M, Roubos EW, Peeters BW, Kozicz T. Sex-dependent and differential responses to acute restraint stress of corticotropin-releasing factor-producing neurons in the rat paraventricular nucleus, central amygdala, and bed nucleus of the stria terminalis. J Neurosci Res. 2012 Jan;90(1):179-92. doi: 10.1002/jnr.22737. Epub 2011 Sep 15. PubMed PMID: 21922520.
198. Sterrenburg L, Gaszner B, Boerrigter J, Santbergen L, Bramini M, Elliott E, Chen A, Peeters BW, Roubos EW, Kozicz T. Chronic stress induces sex-specific alterations in methylation and expression of corticotropin-releasing factor gene in the rat. PLoS One. 2011;6(11):e28128. doi: 10.1371/journal.pone.0028128. Epub 2011 Nov 23. PubMed PMID: 22132228; PubMed Central PMCID: PMC3223222.
199. Suenaga T, Yukie M, Gao S, Nakahara D. Sex-specific effects of prenatal stress on neuronal development in the medial prefrontal cortex and the hippocampus. Neuroreport. 2012 May 9;23(7):430-5. doi:10.1097/WNR.0b013e3283529805. PubMed PMID: 22426025.
200. Sullivan RM, Chehab SL, Dufresne MM, Laplante F. Role of sex in the neurochemical and neuroendocrine correlates of paw preference in the rat. Neuroscience. 2012 Jan 27;202:192-201. doi: 10.1016/j.neuroscience.2011.12.001.Epub 2011 Dec 8. PubMed PMID: 22173010.
201. Sun B, Purcell RH, Terrillion CE, Yan J, Moran TH, Tamashiro KL. Maternal high-fat diet during gestation or suckling differentially affects offspring leptin sensitivity and obesity. Diabetes. 2012 Nov;61(11):2833-41. doi:10.2337/db11-0957. Epub 2012 Jun 29. PubMed PMID: 22751689; PubMed Central PMCID: PMC3478561.
202. Swerdlow NR, Shilling PD, Breier M, Trim RS, Light GA, Marie RS. Fronto-temporal-mesolimbic gene expression and heritable differences in amphetamine-disrupted sensorimotor gating in rats. Psychopharmacology (Berl). 2012 Dec;224(3):349-62. doi: 10.1007/s00213-012-2758-1. Epub 2012 Jun 15. PubMed PMID: 22700037.
203. Sanchez P, Torres JM, del Moral RG, de Dios Luna J, Ortega E. Steroid alpha-reductase in adult rat brain after neonatal testosterone administration. IUBMB Life. 2012 Jan;64(1):81-6. doi: 10.1002/iub.569. Epub 2011 Nov 30. PubMed PMID: 22131296.
204. Takumi K, Iijima N, Ozawa H. Developmental changes in the expression of kisspeptin mRNA in rat hypothalamus. J Mol Neurosci. 2011 Feb;43(2):138-45. doi: 10.1007/s12031-010-9430-1. Epub 2010 Jul 28. PubMed PMID: 20665248.
205. Taraschenko OD, Maisonneuve IM, Glick SD. Sex differences in high fat-induced obesity in rats: Effects of 18-methoxycoronaridine. Physiol Behav. 2011 Jun 1;103(3-4):308-14. doi: 10.1016/j.physbeh.2011.02.011. Epub 2011 Feb 12. PubMed PMID: 21324333.
206. Taylor SB, Taylor AR, Koenig JI. The interaction of disrupted type II neuregulin 1 and chronic adolescent stress on adult anxiety- and fear-related behaviors. Neuroscience. 2013 Sep 26;249:31-42. doi:10.1016/j.neuroscience.2012.09.045. Epub 2012 Sep 25. PubMed PMID: 23022220; PubMed Central PMCID: PMC3568180.
207. Taylor SB, Markham JA, Taylor AR, Kanaskie BZ, Koenig JI. Sex-specific neuroendocrine and behavioral phenotypes in hypomorphic Type II Neuregulin 1 rats. Behav Brain Res. 2011 Oct 31;224(2):223-32. doi: 10.1016/j.bbr.2011.05.008. Epub 2011 May 19. PubMed PMID: 21620900; PubMed Central PMCID: PMC3159843.
208. Thulluri S, Wu M, Blough ER, Manne ND, Litchfield AB, Wang B. Regulation of iron-related molecules in the rat hippocampus: sex- and age-associated differences. Ann Clin Lab Sci. 2012 Spring;42(2):145-51. PubMed PMID: 22585610.
209. Tsuji M, Aoo N, Harada K, Sakamoto Y, Akitake Y, Irie K, Mishima K, Ikeda T, Fujiwara M. Sex differences in the benefits of rehabilitative training during adolescence following neonatal hypoxia-ischemia in rats. Exp Neurol. 2010 Dec;226(2):285-92. doi: 10.1016/j.expneurol.2010.09.002. Epub 2010 Sep 15. PubMed PMID: 20833167.
210. Uban KA, Comeau WL, Ellis LA, Galea LA, Weinberg J. Basal regulation of HPA and dopamine systems is altered differentially in males and females by prenatal alcohol exposure and chronic variable stress. Psychoneuroendocrinology. 2013 Oct;38(10):1953-66. doi: 10.1016/j.psyneuen.2013.02.017. Epub 2013 Apr 8. PubMed PMID: 23579081; PubMed Central PMCID: PMC3758462.
211. van den Bos R, Jolles J, van der Knaap L, Baars A, de Visser L. Male and female Wistar rats differ in decision-making performance in a rodent version of the Iowa Gambling Task. Behav Brain Res. 2012 Oct 1;234(2):375-9. doi:10.1016/j.bbr.2012.07.015. Epub 2012 Jul 16. PubMed PMID: 22814113.
212. Van den Hove DL, Kenis G, Brass A, Opstelten R, Rutten BP, Bruschettini M, Blanco CE, Lesch KP, Steinbusch HW, Prickaerts J. Vulnerability versus resilience to prenatal stress in male and female rats; implications from gene expression profiles in the hippocampus and frontal cortex. Eur Neuropsychopharmacol. 2013 Oct;23(10):1226-46. doi: 10.1016/j.euroneuro.2012.09.011. Epub 2012 Nov 28.PubMed PMID: 23199416.
213. Vassoler FM, White SL, Schmidt HD, Sadri-Vakili G, Pierce RC. Epigenetic inheritance of a cocaine-resistance phenotype. Nat Neurosci. 2013 Jan;16(1):42-7. doi: 10.1038/nn.3280. Epub 2012 Dec 16. PubMed PMID: 23242310; PubMed Central PMCID: PMC3531046.
214. Vetter-O'Hagen CS, Spear LP. The effects of gonadectomy on sex- and age-typical responses to novelty and ethanol-induced social inhibition in adult male and female Sprague-Dawley rats. Behav Brain Res. 2012 Feb 1;227(1):224-32. doi: 10.1016/j.bbr.2011.10.023. Epub 2011 Oct 21. PubMed PMID: 22036699; PubMed Central PMCID: PMC3242866.
215. Vetter-O'Hagen CS, Spear LP. The effects of gonadectomy on age- and sex-typical patterns of ethanol consumption in Sprague-Dawley rats. Alcohol Clin Exp Res. 2011 Nov;35(11):2039-49. doi: 10.1111/j.1530-0277.2011.01555.x. Epub 2011 Jun 8. PubMed PMID: 21651581; PubMed Central PMCID: PMC3170671.
216. Viveros MP, Llorente R, Diaz F, Romero-Zerbo SY, Bermudez-Silva FJ,Rodriguez de Fonseca F, Argente J, Chowen JA. Maternal deprivation has sexually dimorphic long-term effects on hypothalamic cell-turnover, body weight and circulating hormone levels. Horm Behav. 2010 Nov;58(5):808-19. doi: 10.1016/j.yhbeh.2010.08.003. Epub 2010 Aug 11. PubMed PMID: 20708008.
217. Wakley AA, Craft RM. Antinociception and sedation following intracerebroventricular administration of Œî‚Åπ-tetrahydrocannabinol in female vs. male rats. Behav Brain Res. 2011 Jan 1;216(1):200-6. doi:10.1016/j.bbr.2010.07.037. Epub 2010 Aug 6. PubMed PMID: 20692296.
218. Walker AK, Nakamura T, Hodgson DM. Neonatal lipopolysaccharide exposure alters central cytokine responses to stress in adulthood in Wistar rats. Stress. 2010 Nov;13(6):506-15. doi: 10.3109/10253890.2010.489977. Epub 2010 Jul 28.PubMed PMID: 20666652.
219. Walker DM, Kirson D, Perez LF, Gore AC. Molecular profiling of postnatal development of the hypothalamus in female and male rats. Biol Reprod. 2012 Dec 6;87(6):129. doi: 10.1095/biolreprod.112.102798. Print 2012 Jun. PubMed PMID: 23034157.
220. Walls SA, Macklin ZL, Devaud LL. Ethanol-induced loss-of-righting response during ethanol withdrawal in male and female rats: associations with alterations in Arc labeling. Alcohol Clin Exp Res. 2012 Feb;36(2):234-41. doi:10.1111/j.1530-0277.2011.01613.x. Epub 2011 Sep 6. PubMed PMID: 21895716.
221. Wang H, Chen B, Wang Y, Zhou Y. The sexual difference of aging-associated functional degradation in visual cortical cells of rats. Neurosci Lett. 2010 Dec 17;486(3):179-83. doi: 10.1016/j.neulet.2010.09.047. Epub 2010 Sep 22. PubMed PMID: 20868727.
222. Westenbroek C, Perry AN, Becker JB. Pair housing differentially affects motivation to self-administer cocaine in male and female rats. Behav Brain Res. 2013 Sep 1;252:68-71. doi: 10.1016/j.bbr.2013.05.040. Epub 2013 May 30. PubMed PMID: 23727175; PubMed Central PMCID: PMC3742635.
223. Wiley JL, Jones AR, Wright MJ Jr. Exposure to a high-fat diet decreases sensitivity to Œî9-tetrahydrocannabinol-induced motor effects in female rats. Neuropharmacology. 2011 Feb-Mar;60(2-3):274-83. doi:10.1016/j.neuropharm.2010.09.010. Epub 2010 Sep 17. PubMed PMID: 20850461; PubMed Central PMCID: PMC3014410.
224. Williams TJ, Torres-Reveron A, Chapleau JD, Milner TA. Hormonal regulation of delta opioid receptor immunoreactivity in interneurons and pyramidal cells in the rat hippocampus. Neurobiol Learn Mem. 2011 Feb;95(2):206-20. doi:10.1016/j.nlm.2011.01.002. Epub 2011 Jan 9. PubMed PMID: 21224009; PubMed Central PMCID: PMC3045654.
225. Williams TJ, Milner TA. Delta opioid receptors colocalize with corticotropin releasing factor in hippocampal interneurons. Neuroscience. 2011 Apr 14;179:9-22. doi: 10.1016/j.neuroscience.2011.01.034. Epub 2011 Jan 26. PubMed PMID: 21277946; PubMed Central PMCID: PMC3059386.
226. Wilmott LA, Thompson LT. Sex- and dose-dependent effects of post-trial calcium channel blockade by magnesium chloride on memory for inhibitory avoidance conditioning. Behav Brain Res. 2013 Nov 15;257:49-53. doi:10.1016/j.bbr.2013.09.047. Epub 2013 Oct 2. PubMed PMID: 24095881.
227. Wissman AM, May RM, Woolley CS. Ultrastructural analysis of sex differences in nucleus accumbens synaptic connectivity. Brain Struct Funct. 2012 Apr;217(2):181-90. doi: 10.1007/s00429-011-0353-6. Epub 2011 Oct 11. PubMed PMID:21987050; PubMed Central PMCID: PMC3275686.
228. Wissman AM, McCollum AF, Huang GZ, Nikrodhanond AA, Woolley CS. Sex differences and effects of cocaine on excitatory synapses in the nucleus accumbens. Neuropharmacology. 2011 Jul-Aug;61(1-2):217-27. doi: 10.1016/j.neuropharm.2011.04.002. Epub 2011 Apr 13. PubMed PMID: 21510962; PubMed Central PMCID: PMC3105198.
229. Wu X, Kania-Korwel I, Chen H, Stamou M, Dammanahalli KJ, Duffel M, Lein PJ, Lehmler HJ. Metabolism of 2,2',3,3',6,6'-hexachlorobiphenyl (PCB 136) atropisomers in tissue slices from phenobarbital or dexamethasone-induced rats is sex-dependent. Xenobiotica. 2013 Nov;43(11):933-47. doi: 10.3109/00498254.2013.785626. Epub 2013 Apr 12. PubMed PMID: 23581876; PubMedCentral PMCID: PMC3878182.
230. Xanthos DN, P√ºngel I, Wunderbaldinger G, Sandk√ºhler J. Effects of peripheral inflammation on the blood-spinal cord barrier. Mol Pain. 2012 Jun 18;8:44. doi: 10.1186/1744-8069-8-44. PubMed PMID: 22713725; PubMed Central PMCID: PMC3407004.
231. Xiang X, Huang W, Haile CN, Kosten TA. Hippocampal GluR1 associates with behavior in the elevated plus maze and shows sex differences. Behav Brain Res. 2011 Sep 23;222(2):326-31. doi: 10.1016/j.bbr.2011.03.068. Epub 2011 Apr 7. PubMed PMID: 21497621; PubMed Central PMCID: PMC3096704.
232. Xu D, Xia LP, Shen L, Lei YY, Liu L, Zhang L, Magdalou J, Wang H. Prenatal nicotine exposure enhances the susceptibility to metabolic syndrome in adult offspring rats fed high-fat diet via alteration of HPA axis-associated neuroendocrine metabolic programming. Acta Pharmacol Sin. 2013 Dec;34(12):1526-34. doi: 10.1038/aps.2013.171. Epub 2013 Nov 25. PubMed PMID: 24270239.
233. Xu L, Bloem B, Gaszner B, Roubos EW, Kozicz T. Stress-related changes in the activity of cocaine- and amphetamine-regulated transcript and nesfatin neurons in the midbrain non-preganglionic Edinger-Westphal nucleus in the rat. Neuroscience. 2010 Oct 13;170(2):478-88. doi: 10.1016/j.neuroscience.2010.07.001. Epub 2010 Jul 16. PubMed PMID: 20638450.
234. Yang J, Hu LL, Liu LY, Zhao LY, Hou N, Ni L, Li ZF, Wang AY, Song TS, Huang C. Proteomics reveals intersexual differences in the rat brain hippocampus. Anat Rec (Hoboken). 2013 Mar;296(3):462-9. doi: 10.1002/ar.22651. Epub 2013 Feb 5. PubMed PMID: 23381953.
235. Ye Z, Xie Q, Xi G, Keep RF, Hua Y. Effects of gender on heart injury after intracerebral hemorrhage in rats. Acta Neurochir Suppl. 2011;111:119-22. doi: 10.1007/978-3-7091-0693-8_19. PubMed PMID: 21725741.
236. Yee JR, Prendergast BJ. Sex-specific social regulation of inflammatory responses and sickness behaviors. Brain Behav Immun. 2010 Aug;24(6):942-51. doi: 10.1016/j.bbi.2010.03.006. Epub 2010 Mar 17. PubMed PMID: 20303405; PubMed Central PMCID: PMC2897937.
237. Zhang Y, Donica CL, Standifer KM. Sex differences in the Nociceptin/Orphanin FQ system in rat spinal cord following chronic morphine treatment. Neuropharmacology. 2012 Sep;63(3):427-33. doi: 10.1016/j.neuropharm.2012.04.028. Epub 2012 May 2. PubMed PMID: 22575074.
238. Zhou L, Ghee SM, Chan C, Lin L, Cameron MD, Kenny PJ, See RE. Orexin-1 receptor mediation of cocaine seeking in male and female rats. J Pharmacol Exp Ther. 2012 Mar;340(3):801-9. doi: 10.1124/jpet.111.187567. Epub 2011 Dec 20. PubMed PMID: 22186370; PubMed Central PMCID: PMC3286310.
239. Zitman FM, Richter-Levin G. Age and sex-dependent differences in activity, plasticity and response to stress in the dentate gyrus. Neuroscience. 2013 Sep 26;249:21-30. Doi: 10.1016/j.neuroscience.2013.05.030. Epub 2013 May 29. PubMed PMID: 23727507.

**Web of Science References Used:**

1. Adamek S, Vyskocil F. Potassium-selective microelectrode revealed difference in threshold potassium concentration for cortical spreading depression in female and male rat brain. Brain Research. 2011;1370:215-9. doi: 10.1016/j.brainres.2010.11.018. PubMed PMID: WOS:000287065900020.
2. Aksu I, Baykara B, Ozbal S, Cetin F, Sisman AR, Dayi A, et al. Maternal treadmill exercise during pregnancy decreases anxiety and increases prefrontal cortex VEGF and BDNF levels of rat pups in early and late periods of life. Neuroscience Letters. 2012;516(2):221-5. doi: 10.1016/j.neulet.2012.03.091. PubMed PMID: WOS:000304520300012.
3. Anderson ML, Nokia MS, Govindaraju KP, Shors TJ. MODERATE DRINKING? ALCOHOL CONSUMPTION SIGNIFICANTLY DECREASES NEUROGENESIS IN THE ADULT HIPPOCAMPUS. Neuroscience. 2012;224:202-9. doi:
4. Bai M, Zhang L, Zhu XZ, Zhang Y, Zhang S, Xue L. Comparison of depressive behaviors induced by three stress paradigms in rats. Physiology & Behavior. 2014;131:81-6. doi: 10.1016/j.physbeh.2014.04.019. PubMed PMID: WOS:000337866600012.
5. Berquist MD, Mooney-Leber SM, Feifel D, Prus AJ. Assessment of attention in male and female Brattleboro rats using a self-paced five-choice serial reaction time task. Brain Research. 2013;1537:174-9. doi: 10.1016/j.brainres.2013.09.012. PubMed PMID: WOS:000327915200019.
6. Bertholet L, Meunier C, Preissmann D, Schenk F. Sex biased spatial strategies relying on the integration of multimodal cues in a rat model of schizophrenia: Impairment in predicting future context? Behavioural Brain Research. 2014;262:109-17. doi: 10.1016/j.bbr.2013.12.039. PubMed PMID: WOS:000332356400015.
7. Bessalova EY. Behavioral Activity and Anxiety of Rats under Open Field Conditions in the Norm and after Parenteral Introductions of Xenogenic Cerebrospinal Fluid. Neurophysiology. 2012;43(5):369-76. PubMed PMID: WOS:000299536500006.
8. Bessinis DP, Dalla C, Kokras N, Pitychoutis PM, Papadopoulou-Daifoti Z. SEX-DEPENDENT NEUROCHEMICAL EFFECTS OF ENVIRONMENTAL ENRICHMENT IN THE VISUAL SYSTEM. Neuroscience. 2013;254:130-40. doi: 10.1016/j.neuroscience.2013.09.013. PubMed PMID: WOS:000327279400012.
9. Blaze J, Roth TL. Exposure to caregiver maltreatment alters expression levels of epigenetic regulators in the medial prefrontal cortex. International Journal of Developmental Neuroscience. 2013;31(8):804-10. doi: 10.1016/j.ijdevneu.2013.10.001. PubMed PMID: WOS:000329270200012.
10. Brocardo PS, Boehme F, Patten A, Cox A, Gil-Mohapel J, Christie BR. Anxiety- and depression-like behaviors are accompanied by an increase in oxidative stress in a rat model of fetal alcohol spectrum disorders: Protective effects of voluntary physical exercise. Neuropharmacology. 2012;62(4):1607-18. doi: 10.1016/j.neuropharm.2011.10.006. PubMed PMID: WOS:000301221500001.
11. Bruner NR, Salm AK, Anderson KG. Effects of prenatal stress on lever-press acquisition with delayed reinforcement in male and female rats. Behavioural Processes. 2012;89(3):256-63. doi: 10.1016/j.beproc.2011.12.003. PubMed PMID: WOS:000301886000009.
12. Brydges NM, Wood ER, Holmes MC, Hall J. Prepubertal stress and hippocampal function: Sex-specific effects. Hippocampus. 2014;24(6):684-92. doi: 10.1002/hipo.22259. PubMed PMID: WOS:000335945900008.
13. Cambras T, Castejon L, Diez-Noguera A. Social interaction and sex differences influence rat temperature circadian rhythm under LD cycles and constant light. Physiology & Behavior. 2011;103(3-4):365-71. doi: 10.1016/j.physbeh.2011.03.010. PubMed PMID: WOS:000291294000017.
14. Caruso D, Pesaresi M, Abbiati F, Calabrese D, Giatti S, Garcia-Segura LM, et al. Comparison of plasma and cerebrospinal fluid levels of neuroactive steroids with their brain, spinal cord and peripheral nerve levels in male and female rats. Psychoneuroendocrinology. 2013;38(10):2278-90. doi: 10.1016/j.psyneuen.2013.04.016. PubMed PMID: WOS:000326213700034.
15. Catak Z, Aydin S, Sahin I, Kuloglu T, Aksoy A, Dagli AF. Regulatory neuropeptides (ghrelin, obestatin and nesfatin-1) levels in serum and reproductive tissues of female and male rats with fructose-induced metabolic syndrome. Neuropeptides. 2014;48(3):167-77. doi: 10.1016/j.npep.2014.04.002. PubMed PMID: WOS:000336825000007.
16. Chen J, Evans AN, Liu Y, Honda M, Saavedra JM, Aguilera G. Maternal Deprivation in Rats is Associated with Corticotrophin-Releasing Hormone (CRH) Promoter Hypomethylation and Enhances CRH Transcriptional Responses to Stress in Adulthood. Journal of Neuroendocrinology. 2012;24(7):1055-64. doi: 10.1111/j.1365-2826.2012.02306.x. PubMed PMID: WOS:000305453900008.
17. Chocyk A, Przyborowska A, Dudys D, Majcher I, Mackowiak M, Wedzony K. THE IMPACT OF MATERNAL SEPARATION ON THE NUMBER OF TYROSINE HYDROXYLASE-EXPRESSING MIDBRAIN NEURONS DURING DIFFERENT STAGES OF ONTOGENESIS. Neuroscience. 2011;182:43-61. doi: 10.1016/j.neuroscience.2011.03.008. PubMed PMID: WOS:000290460600004.
18. Craft RM, Kandasamy R, Davis SM. Sex differences in anti-allodynic, anti-hyperalgesic and anti-edema effects of Delta(9)-tetrahydrocannabinol in the rat. Pain. 2013;154(9):1709-17. doi: 10.1016/j.pain.2013.05.017. PubMed PMID: WOS:000323600000028.
19. 3Diehl LA, Pereira NDC, Laureano DP, Benitz AND, Noschang C, Ferreira AGK, et al. Contextual Fear Conditioning in Maternal Separated Rats: The Amygdala as a Site for Alterations. Neurochemical Research. 2014;39(2):384-93. doi: 10.1007/s11064-013-1230-x. PubMed PMID: WOS:000330839200019.
20. Farrell MR, Sengelaub DR, Wellman CL. Sex differences and chronic stress effects on the neural circuitry underlying fear conditioning and extinction. Physiology & Behavior. 2013;122:208-15. doi: 10.1016/j.physbeh.2013.04.002. PubMed PMID: WOS:000329552700031.
21. Ferguson SA, Berry KJ. Chronic oral treatment with isotretinoin alters measures of activity but not anxiety in male and female rats. Neurotoxicology and Teratology. 2010;32(5):573-8. doi: 10.1016/j.ntt.2010.03.009. PubMed PMID: WOS:000281940300008.
22. Ferguson SA, Law CD, Abshire JS. Developmental treatment with bisphenol A causes few alterations on measures of postweaning activity and learning. Neurotoxicology and Teratology. 2012;34(6):598-606. doi: 10.1016/j.ntt.2012.09.006. PubMed PMID: WOS:000312518600008.
23. Ferrari LF, Bogen O, Levine JD. Role of Nociceptor alpha CaMKII in Transition from Acute to Chronic Pain (Hyperalgesic Priming) in Male and Female Rats. Journal of Neuroscience. 2013;33(27):11002-11. doi: 10.1523/jneurosci.1785-13.2013. PubMed PMID: WOS:000321258000007.
24. Ferrari LF, Bogen O, Levine JD. Second Messengers Mediating the Expression of Neuroplasticity in a Model of Chronic Pain in the Rat. Journal of Pain. 2014;15(3):312-20. doi: 10.1016/j.jpain.2013.12.005. PubMed PMID: WOS:000333437800012.
25. Filipovic N, Zuvan L, Masek T, Tokalic R, Grkovic I. Gender and gonadectomy influence on neurons in superior cervical ganglia of sexually mature rats. Neuroscience Letters. 2014;563:55-60. doi: 10.1016/j.neulet.2014.01.028. PubMed PMID: WOS:000334005900012.
26. Goel N, Innala L, Viau V. Sex differences in serotonin (5-HT) 1A receptor regulation of HPA axis and dorsal raphe responses to acute restraint. Psychoneuroendocrinology. 2014;40:232-41. doi: 10.1016/j.psyneuen.2013.11.020. PubMed PMID: WOS:000331921300026.
27. Gomez ML, Martinez-Mota L, Estrada-Camarena E, Fernandez-Guasti A. INFLUENCE OF THE BRAIN SEXUAL DIFFERENTIATION PROCESS ON DESPAIR AND ANTIDEPRESSANT-LIKE EFFECT OF FLUOXETINE IN THE RAT FORCED SWIM TEST. Neuroscience. 2014;261:11-22. doi: 10.1016/j.neuroscience.2013.12.035. PubMed PMID: WOS:000331095400002.
28. Higuera-Matas A, Botreau F, Del Olmo N, Miguens M, Olias O, Montoya GL, et al. Periadolescent exposure to cannabinoids alters the striatal and hippocampal dopaminergic system in the adult rat brain. European Neuropsychopharmacology. 2010;20(12):895-906. doi: 10.1016/j.euroneuro.2010.06.017. PubMed PMID: WOS:000284569600008.
29. Holden JE, Wang E, Moes JR, Wagner M, Maduko A, Jeong Y. DIFFERENCES IN CARBACHOL DOSE, PAIN CONDITION, AND SEX FOLLOWING LATERAL HYPOTHALAMIC STIMULATION. Neuroscience. 2014;270:226-35. doi: 10.1016/j.neuroscience.2014.04.020. PubMed PMID: WOS:000336624000022.
30. Howland JG, Cazakoff BN, Zhang Y. ALTERED OBJECT-IN-PLACE RECOGNITION MEMORY, PREPULSE INHIBITION, AND LOCOMOTOR ACTIVITY IN THE OFFSPRING OF RATS EXPOSED TO A VIRAL MIMETIC DURING PREGNANCY. Neuroscience. 2012;201:184-98. doi: 10.1016/j.neuroscience.2011.11.011. PubMed PMID: WOS:000299400700017.
31. Hruba L, Schutova B, Slamberova R. Sex differences in anxiety-like behavior and locomotor activity following prenatal and postnatal methamphetamine exposure in adult rats. Physiology & Behavior. 2012;105(2):364-70. doi: 10.1016/j.physbeh.2011.08.016. PubMed PMID: WOS:000300459800031.
32. Joseph EK, Levine JD. Sexual dimorphism in endothelin-1 induced mechanical hyperalgesia in the rat. Experimental Neurology. 2012;233(1):505-12. doi: 10.1016/j.expneurol.2011.11.030. PubMed PMID: WOS:000300123900056.
33. Kesby JP, O'Loan JC, Alexander S, Deng C, Huang XF, McGrath JJ, et al. Developmental vitamin D deficiency alters MK-801-induced behaviours in adult offspring. Psychopharmacology. 2012;220(3):455-63. doi: 10.1007/s00213-011-2492-0. PubMed PMID: WOS:000301545500002.
34. Krugers HJ, Oomen CA, Gumbs M, Li MH, Velzing EH, Joels M, et al. Maternal deprivation and dendritic complexity in the basolateral amygdala. Neuropharmacology. 2012;62(1):534-7. doi: 10.1016/j.neuropharm.2011.09.022. PubMed PMID: WOS:000296826800058.
35. Ma B, Yu LH, Fan JA, Cong BH, He P, Ni X, et al. Estrogen modulation of peripheral pain signal transduction: involvement of P2X(3) receptors. Purinergic Signalling. 2011;7(1):73-83. doi: 10.1007/s11302-010-9212-9. PubMed PMID: WOS:000288804900007.
36. Martinez-Mota L, Ulloa RE, Herrera-Perez J, Chavira R, Fernandez-Guasti A. Sex and age differences in the impact of the forced swimming test on the levels of steroid hormones. Physiology & Behavior. 2011;104(5):900-5. doi: 10.1016/j.physbeh.2011.05.027. PubMed PMID: WOS:000296208200034.
37. McFadden L, Yamamoto BK, Matuszewich L. Alterations in adult behavioral responses to cocaine and dopamine transporters following juvenile exposure to methamphetamine. Behavioural Brain Research. 2011;216(2):726-30. doi: 10.1016/j.bbr.2010.08.041. PubMed PMID: WOS:000285217300035.
38. McFadden LM, Carter S, Matuszewich L. Juvenile exposure to methamphetamine attenuates behavioral and neurochemical responses to methamphetamine in adult rats. Behavioural Brain Research. 2012;229(1):118-22. doi: 10.1016/j.bbr.2012.01.013. PubMed PMID: WOS:000302047600014.
39. McFadden LM, Paris JJ, Mitzelfelt MS, McDonough S, Frye CA, Matuszewich L. Sex-dependent effects of chronic unpredictable stress in the water maze. Physiology & Behavior. 2011;102(3-4):266-75. doi: 10.1016/j.physbeh.2010.10.022. PubMed PMID: WOS:000287423400003.
40. Mohagheghi F, Ahmadiani A, Rahmani B, Moradi F, Romond N, Khalaj L. Gemfibrozil Pretreatment Resulted in a Sexually Dimorphic Outcome in the Rat Models of Global Cerebral Ischemia-Reperfusion via Modulation of Mitochondrial Pro-survival and Apoptotic Cell Death Factors as well as MAPKs. Journal of Molecular Neuroscience. 2013;50(3):379-93. doi: 10.1007/s12031-012-9932-0. PubMed PMID: WOS:000320048400001.
41. Mychasiuk R, Muhammad A, Gibb R, Kolb B. Long-term alterations to dendritic morphology and spine density associated with prenatal exposure to nicotine. Brain Research. 2013;1499:53-60. doi: 10.1016/j.brainres.2012.12.021. PubMed PMID: WOS:000316710300006.
42. Naslund J, Studer E, Nilsson K, Westberg L, Eriksson E. Serotonin depletion counteracts sex differences in anxiety-related behaviour in rat. Psychopharmacology. 2013;230(1):29-35. doi: 10.1007/s00213-013-3133-6. PubMed PMID: WOS:000325700700005.
43. Negrigo A, Medeiros M, Guinsburg R, Covolan L. Long-term gender behavioral vulnerability after nociceptive neonatal formalin stimulation in rats. Neuroscience Letters. 2011;490(3):196-9. doi: 10.1016/j.neulet.2010.12.050. PubMed PMID: WOS:000287794700008.
44. Nicotra L, Tuke J, Grace PM, Rolan PE, Hutchinson MR. Sex differences in mechanical allodynia: how can it be preclinically quantified and analyzed? Frontiers in Behavioral Neuroscience. 2014;8. doi: 10.3389/fnbeh.2014.00040. PubMed PMID: WOS:000331626800001.
45. Niu KY, Ro JY. Changes in intramuscular cytokine levels during masseter inflammation in male and female rats. Neuroscience Letters. 2011;487(2):223-7. doi: 10.1016/j.neulet.2010.10.027. PubMed PMID: WOS:000286793100021.
46. Nosenko ND, Sinitsyn PV, Reznikov AG. Role of Calcium Signaling in the Development of Prenatal Stress-Induced Functional Modifications of the Hypothalamo-Pituitary-Adrenal Axis. Neurophysiology. 2011;42(4):251-7. doi: 10.1007/s11062-011-9157-9. PubMed PMID: WOS:000286437600004.
47. Patel DA, Booze RM, Mactutus CF. Prenatal cocaine exposure alters progenitor cell markers in the subventricular zone of the adult rat brain. International Journal of Developmental Neuroscience. 2012;30(1):1-9. doi: 10.1016/j.ijdevneu.2011.11.001. PubMed PMID: WOS:000299982500001.
48. Patten AR, Brocardo PS, Sakiyama C, Wortman RC, Noonan A, Gil-Mohapel J, et al. Impairments in Hippocampal Synaptic Plasticity following Prenatal Ethanol Exposure are Dependent on Glutathione Levels. Hippocampus. 2013;23(12):1463-75. doi: 10.1002/hipo.22199. PubMed PMID: WOS:000327157200029.
49. Qiu X, Huang CX, Lu W, Yang S, Li C, Shi XY, et al. Effects of a 4 month enriched environment on the hippocampus and the myelinated fibers in the hippocampus of middle-aged rats. Brain Research. 2012;1465:26-33. doi: 10.1016/j.brainres.2012.05.025. PubMed PMID: WOS:000306448300004.
50. Qiu X, Li C, Jiang R, Chen L, Huang CX, Yang S, et al. The effects of short-term enriched environment on capillaries of the middle-aged rat cortex. Neuroscience Letters. 2011;505(2):186-90. doi: 10.1016/j.neulet.2011.10.019. PubMed PMID: WOS:000297614600026.
51. Raftogianni A, Stamatakis A, Diamantopoulou A, Kollia AM, Stylianopoulou F. EFFECTS OF AN EARLY EXPERIENCE OF REWARD THROUGH MATERNAL CONTACT OR ITS DENIAL ON THE DOPAMINERGIC SYSTEM OF THE RAT BRAIN. Neuroscience. 2014;269:11-20. doi: 10.1016/j.neuroscience.2014.03.035. PubMed PMID: WOS:000335903900002.
52. Richards S, Mychasiuk R, Kolb B, Gibb R. Tactile stimulation during development alters behaviour and neuroanatomical organization of normal rats. Behavioural Brain Research. 2012;231(1):86-91. doi: 10.1016/j.bbr.2012.02.043. PubMed PMID: WOS:000304024900009.
53. Ryan CL, Robbins MA, Smith MT, Gallant IC, Adams-Marriott AL, Doucette TA. Altered social interaction in adult rats following neonatal treatment with domoic acid. Physiology & Behavior. 2011;102(3-4):291-5. doi: 10.1016/j.physbeh.2010.11.020. PubMed PMID: WOS:000287423400006.
54. Sabihi S, Durosko NE, Dong SM, Leuner B. Oxytocin in the prelimbic medial prefrontal cortex reduces anxiety-like behavior in female and male rats. Psychoneuroendocrinology. 2014;45:31-42. doi: 10.1016/j.psyneuen.2014.03.009. PubMed PMID: WOS:000337208900004.
55. Salas-Ramirez KY, Frankfurt M, Alexander A, Luine VN, Friedman E. PRENATAL COCAINE EXPOSURE INCREASES ANXIETY, IMPAIRS COGNITIVE FUNCTION AND INCREASES DENDRITIC SPINE DENSITY IN ADULT RATS: INFLUENCE OF SEX. Neuroscience. 2010;169(3):1287-95. doi: 10.1016/j.neuroscience.2010.04.067. PubMed PMID: WOS:000281109200032.
56. Schroeder M, Weller A. Anxiety-like behavior and locomotion in CCK1 knockout rats as a function of strain, sex and early maternal environment. Behavioural Brain Research. 2010;211(2):198-207. doi: 10.1016/j.bbr.2010.03.038. PubMed PMID: WOS:000278168200009.
57. Suenaga T, Yukie M, Gao SB, Nakahara D. Sex-specific effects of prenatal stress on neuronal development in the medial prefrontal cortex and the hippocampus. Neuroreport. 2012;23(7):430-5. doi: 10.1097/WNR.0b013e3283529805. PubMed PMID: WOS:000302948500005.
58. 131. Tekes K, Szegi P, Laufer R, Hantos M, Csaba G. Effect of perinatal stress on the biogenic amine neurotransmitter level of the adult rat's brain. International Journal of Developmental Neuroscience. 2011;29(2):171-5. doi: 10.1016/j.ijdevneu.2010.11.004. PubMed PMID: WOS:000288408200009.
59. Tien LT, Cai ZW, Rhodes PG, Fan LW. Neonatal exposure to lipopolysaccharide enhances methamphetamine-induced reinstated behavioral sensitization in adult rats. Behavioural Brain Research. 2011;224(1):166-73. doi: 10.1016/j.bbr.2011.05.038. PubMed PMID: WOS:000293312200023.
60. Torres-Chavez KE, Sanfins JM, Clemente-Napimoga JT, Pelegrini-Da-Silva A, Parada CA, Fischer L, et al. Effect of gonadal steroid hormones on formalin-induced temporomandibular joint inflammation. European Journal of Pain. 2012;16(2):204-16. doi: 10.1016/j.ejpain.2011.06.007. PubMed PMID: WOS:000306900900006.
61. Van den Hove DLA, Leibold NK, Strackx E, Martinez-Claros M, Lesch KP, Steinbusch HWM, et al. Prenatal stress and subsequent exposure to chronic mild stress in rats; interdependent effects on emotional behavior and the serotonergic system. European Neuropsychopharmacology. 2014;24(4):595-607. doi: 10.1016/j.euroneuro.2013.09.006. PubMed PMID: WOS:000334136000013.
62. Vatanparast J, Naseh M, Baniasadi M, Haghdoost-Yazdi H. Developmental exposure to chlorpyrifos and diazinon differentially affect passive avoidance performance and nitric oxide synthase-containing neurons in the basolateral complex of the amygdala. Brain Research. 2013;1494:17-27. doi: 10.1016/j.brainres.2012.11.049. PubMed PMID: WOS:000314559700003.
63. Victoria NC, Inouec K, Young LJ, Murphy AZ. A Single Neonatal Injury Induces Life-Long Deficits in Response to Stress. Developmental Neuroscience. 2013;35(4):326-37. doi: 10.1159/000351121. PubMed PMID: WOS:000323857000005.
64. Villa RF, Ferrari F, Gorini A. ENERGY METABOLISM OF RAT CEREBRAL CORTEX, HYPOTHALAMUS AND HYPOPHYSIS DURING AGEING. Neuroscience. 2012;227:55-66. doi: 10.1016/j.neuroscience.2012.09.041. PubMed PMID: WOS:000312113100006.
65. Virdee K, McArthur S, Brischoux F, Caprioli D, Ungless MA, Robbins TW, et al. Antenatal Glucocorticoid Treatment Induces Adaptations in Adult Midbrain Dopamine Neurons, which Underpin Sexually Dimorphic Behavioral Resilience. Neuropsychopharmacology. 2014;39(2):339-50. doi: 10.1038/npp.2013.196. PubMed PMID: WOS:000328585400011.
66. Weston HI, Sobolewski ME, Allen JL, Weston D, Conrad K, Pelkowski S, et al. Sex-dependent and non-monotonic enhancement and unmasking of methylmercury neurotoxicity by prenatal stress. Neurotoxicology. 2014;41:123-40. doi: 10.1016/j.neuro.2014.01.009. PubMed PMID: WOS:000334092200013.
67. Yamamotova A, Hruba L, Schutova B, Rokyta R, Slamberova R. Perinatal effect of methamphetamine on nociception in adult Wistar rats. International Journal of Developmental Neuroscience. 2011;29(1):85-92. doi: 10.1016/j.ijdevneu.2010.08.004. PubMed PMID: WOS:000286846500012.
68. Zhang HY, Fan YS, Xia F, Geng CS, Mao CP, Jiang S, et al. Prenatal water deprivation alters brain angiotensin system and dipsogenic changes in the offspring. Brain Research. 2011;1382:128-36. doi: 10.1016/j.brainres.2011.01.031. PubMed PMID: WOS:000289135600014.
69. Zhang JL, Dennis KA, Darling RD, Alzghoul L, Paul IA, Simpson KL, et al. Neonatal citalopram exposure decreases serotonergic fiber density in the olfactory bulb of male but not female adult rats. Frontiers in Cellular Neuroscience. 2013;7. doi: 10.3389/fncel.2013.00067. PubMed PMID: WOS:000318843700001.
70. Zhang X, Zhang Y, Asgar J, Niu KY, Lee J, Lee KS, et al. Sex differences in mu-opioid receptor expression in trigeminal ganglia under a myositis condition in rats. European Journal of Pain. 2014;18(2):151-61. doi: 10.1002/j.1532-2149.2013.00352.x. PubMed PMID: WOS:000329303600002.
71. Zhang Y, Cazakoff BN, Thai CA, Howland JG. Prenatal exposure to a viral mimetic alters behavioural flexibility in male, but not female, rats. Neuropharmacology. 2012;62(3):1299-307. doi: 10.1016/j.neuropharm.2011.02.022. PubMed PMID: WOS:000300533800015.
72. Zhang Y, Lu N, Zhao ZQ, Zhang YQ. Involvement of Estrogen in Rapid Pain Modulation in the Rat Spinal Cord. Neurochemical Research. 2012;37(12):2697-705. doi: 10.1007/s11064-012-0859-1. PubMed PMID: WOS:000311537300006.
